# Activation Energy of Organic Matter Decomposition in Soil and Consequences of Global Warming

**DOI:** 10.1111/gcb.70472

**Published:** 2025-09-04

**Authors:** Ekaterina Filimonenko, Yakov Kuzyakov

**Affiliations:** ^1^ National Key Laboratory of Wheat Improvement College of Agronomy, Shandong Agricultural University Tai'an Shandong China; ^2^ Sirius University of Science and Technology, Sirius Federal Area Sochi Russia; ^3^ Department of Soil Science of Temperate Ecosystems, Department of Agricultural Soil Science University of Gottingen Gottingen Germany; ^4^ Peoples Friendship University of Russia (RUDN University) Moscow Russia

**Keywords:** carbon and nutrient cycles, energy barrier, microbial activities, microcalorimetry, oxidative and hydrolytic enzymes, soil organic matter stability, soil thermal analysis

## Abstract

The activation energy (*E*_*a*_) is the minimum energy necessary for (bio)chemical reactions acting as an energy barrier and defining reaction rates, for example, organic matter transformations in soil. Based on the *E*_*a*_ database of (i) oxidative and hydrolytic enzyme activities, (ii) organic matter mineralization and CO_2_ production, (iii) heat release during soil incubation, as well as (iv) thermal oxidation of soil organic matter (SOM), we assess the *E*_*a*_ of SOM transformation processes. After a short description of the four approaches to assess these *E*_*a*_ values—all based on the Arrhenius equation—we present the *E*_*a*_ of chemical oxidation (79 kJ mol^−1^, based on thermal oxidation), microbial mineralization (67 kJ mol^−1^, CO_2_ production), microbial decomposition (40 kJ mol^−1^, heat release), and enzyme‐catalyzed hydrolysis of polymers and cleavage of mineral ions of nutrients (33 kJ mol^−1^, enzyme driven reactions) from SOM. The catalyzing effects of hydrolytic and oxidative enzymes reduce *E*_*a*_ of SOM decomposition by more than twice that of its chemical oxidation. The *E*_*a*_ of enzymatic cleavage of mineral ions of N, P, and S from their organic compounds is 9 kJ mol^−1^ lower (corresponding to 40‐fold faster reactions) than the hydrolysis of N‐, P‐, and S‐free organic polymers. In soil, where organic compounds are physically protected and enzymes are partly deactivated, microbial mineralization is ~140‐fold faster compared to its pure chemical oxidation. Because processes with higher *E*_*a*_ are more sensitive to temperature increase, global warming will accelerate the decomposition of stable organic compounds and boost the C cycle much stronger than the cycling of nutrients: N, P, and S. Consequently, the stoichiometry of microbially utilized compounds in warmer conditions will shift toward organic pools with higher C/N ratios. This will decouple the cycling of C and nutrients: N, P, and S. Overall, the *E*_*a*_ of (bio)chemical transformations of organic matter in soil enables to assess process rates and the inherent stability of SOM pools, as well as their responses to global warming.

## Graphical abstract

Activation energy (*E*_*a*_) is the minimum energy necessary for (bio)chemical reactions defining reaction rates, e.g., organic matter transformations in soil. Based on the *E*_*a*_ database of enzyme activities, CO_2_ production, heat release during soil incubation, and thermal oxidation of soil organic matter, we assess the *E*_*a*_ of biotic and abiotic transformation processes in soil. Because processes with higher *E*_*a*_ are more sensitive to temperature increase, global warming will accelerate the decomposition of stable organic compounds and boost the C cycle much stronger than the cycling of nutrients: N, P, and S.

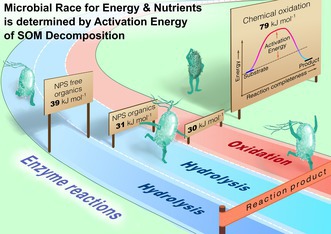

## 1 Introduction

**FIGURE 1:**
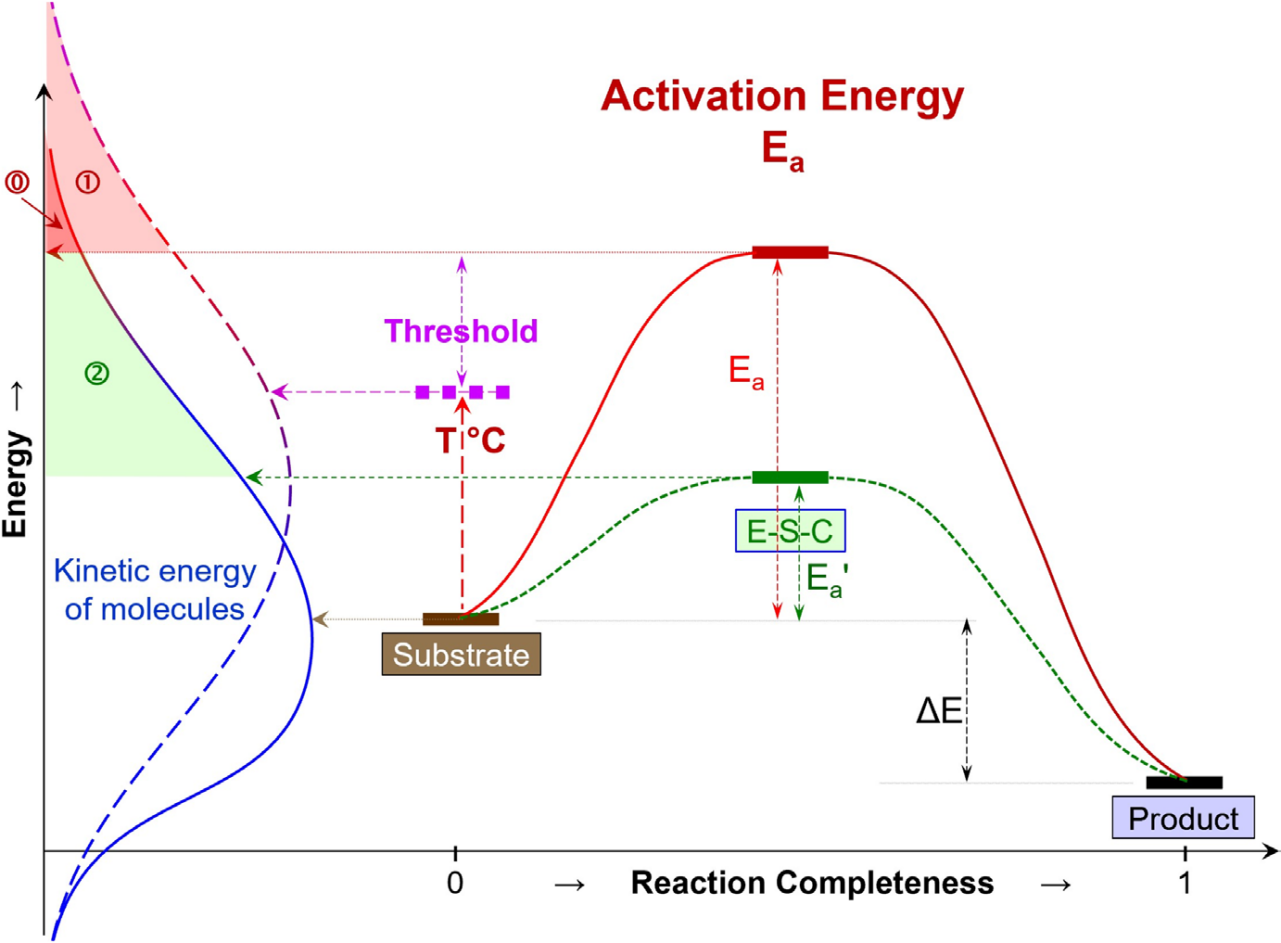
The concept of activation energy (*E*_*a*_), the corresponding kinetic energy of molecules (left), and two options to accelerate the reaction rate: (1) temperature increase, (2) change of the reaction mechanism, e.g., by a catalyst. Effects of the energy of reactant molecules to overcome the energy barrier of a reaction: ⓪ under normal temperature, ① rising temperature (red shaded area, left) increases the number of reactant molecules with sufficient energy for the reaction, and ② by catalysts, (e.g., by enzymes). Reducing the activation energy (by modifying the reaction mechanisms, e.g., by enzymes) increases the reaction probability and the rate because the energy of more molecules equals or is above the height of the energy barrier. *E*_*a*_: Activation energy (red: Initial reaction, green: Modified reaction); *T* °C: Temperature increase of the substrate, thus decreasing the threshold to the *E*_*a*_; E‐S‐C: Enzyme‐Substrate‐Complex; Δ*E*: Difference in the enthalpy between the substrate and product, frequently released as heat. Threshold: The energy necessary for the molecules to reach the activation energy.

Decomposition rates of soil organic matter (SOM) are defined by its spatial accessibility to microorganisms and enzymes, as well as by the biochemical stability of organic compounds and their associations with clay minerals and metal oxides (Conant et al. 2011; von Lützow et al. 2006; Sollins et al. 1996). The SOM transformation by chemical and biochemical processes can occur when reactant molecules have an energy equal to or greater than the activation energy (*E*_*a*_) of reaction (Box 1). The widely used concept of *E*_*a*_ as the energy barrier (Figure 1) provides insight into the stability of organic compounds—the required energy of molecules for a reaction. Despite a simplification (Menzinger and Wolfgang 1969) and assumptions in evaluations of biological processes (Alster et al. 2022; Hobbs et al. 2013; Schipper et al. 2014), the *E*_*a*_ is specific to a reaction and remains constant when the underlying mechanism remains unaltered.

BOX 1 **Background Definitions.**

**Activation energy (*E*_*a*_)** is the minimum amount of energy that must be available for the molecules of reactants to initiate a chemical reaction (Figure 1). *E*_*a*_ is determined by the reaction mechanism and does not depend on temperature. *E*_*a*_ of a reaction is measured in kilojoules per mole (kJ mol^−1^).

**Activation energy (*E***_***a***_ **) of SOM decomposition** is a measure of the energy required to decompose SOM. The *E*_*a*_ of SOM decomposition can be used as a proxy for the inherent stability of the SOM.

**Arrhenius equation** is an empirical formula for the rate constant of a chemical reaction (*k*) depending on the absolute temperature:

$$k\left(T\right)=A\times e^{\frac{-E_{a}}{R\times T}}$$

where *A* is the pre‐exponential factor (s^−1^); *E*_*a*_ is the activation energy (kJ mol^−1^); R is the universal gas constant (8.314 J K^−1^ mol^−1^); and *T* is absolute temperature (K).

**Energy barrier** is the energy that reactant molecules must exceed to initiate the reaction. The energy barrier is not necessarily equal to the *E*_*a*_ because the temperature of the substrates may differ from the standard conditions (25°C, 1.0 atm pressure). In this paper, however, we use energy barrier as a synonym for *E*_*a*_.

**Enthalpy** is the total energy of heat in the system, which is equivalent to the sum of total internal energy and resulting energy due to its pressure and volume changes. The system has internal energy both because of the molecules in motion and the state of molecules. Because soil is an open system (constant pressure and volume), the entropy change during the processes of soil organic matter transformation within incubation experiments can be neglected due to extremely low values. Consequently, changes of enthalpy are identical to changes of Gibbs energy.

**Hydrolysis** is the cleavage of molecules by reaction with water. A water molecule is consumed to cleave a larger molecule into component parts. CO_2_ is not produced by hydrolytic reactions, but hydrolysis releases a large amount of energy partly as a heat flux. Accordingly, caution should be exercised when directly comparing CO_2_ respired by soil incubations with heat release from SOM decomposition (calorespirometric ratio).

**Return‐on‐Investment** (ROI) is a concept that the amount of energy obtained by a microbial community (or a microbial cell), e.g., from the outcome of enzymatic reactions, should be larger than the energy they invested for enzyme production. Despite the attractive concept, both the energy investment and the energy yield cannot be easily quantified: The energy investment for enzyme production by a cell is not known. This energy investment does not correspond and has nothing to do with the *E*_*a*_ of the reaction catalyzed by the enzyme (Figure 1). The energy returned is also not known (does not correspond to Δ*E* in Figure 1) because the number of the catalyzed reactions over the enzyme life‐time is also unknown. Furthermore, the losses and deactivation of the enzyme in soil and the products of the enzymatic reaction before the uptake by a microbial cell are not known. So, despite the attractiveness of the ROI concept from the view of microbial adaptations and evolution, it cannot be calculated based on the *E*_*a*_ and Δ*E* in Figure 1.

**SOM decomposition** is the sum of chemical and biological processes, which involve chemical breakdown and biochemical transformation of organic materials into simple organic and inorganic molecules. SOM decomposition is a stage of SOM mineralization.

**SOM mineralization** is the sum of chemical and biological processes of complete decomposition, by which carbon and hydrogen are completely oxidized to CO_2_ and H_2_O, and nutrients are transformed into plant‐available mineral forms of nitrogen, phosphorus, and sulfur.

**SOM stability** is an inherent property of SOM, determined by its structural and molecular composition as well as by binding on clays and short‐range ordered minerals. It reflects decomposability regardless of the mechanisms (chemical or biological). We assume here that SOM stability increases with the *E*_*a*_ necessary for its decomposition (hydrolysis and/or mineralization).

**SOM transformation** is used here to indicate changes of SOM not necessarily leading to decomposition or complete mineralization.

There are two ways to overcome the energy barrier of a reaction in soils: (i) increase the probability of reaction by raising the temperature, which increases the number of reactant molecules with sufficient energy (Kleber 2010); or (ii) reduce the activation energy by modifying the reaction mechanism, e.g., by a catalyst (Figure 1). An increase in pressure, which is the 3rd potential mechanism to overcome the energy barrier for reactions in gases, is not relevant for the reactions in soils.

SOM dynamics are largely controlled by SOM stabilization and destabilization mechanisms (Sollins et al. 1996; von Lützow et al. 2006). Stabilization involves three key processes that reduce the accessibility of organic matter to microbial decomposition: (i) chemical recalcitrance of organic compounds; (ii) occlusion, intercalation, hydrophobicity, and encapsulation of organic material; and (iii) interactions with mineral surfaces and polyvalent metal ions. Despite being suggested nearly 30 years ago (Sollins et al. 1996) and evaluated in many subsequent studies and reviews (Conant et al. 2011; von Lützow et al. 2006), these studies did not provide quantitative parameters of stabilization by recalcitrance, occlusion, intercalation, and encapsulation (except hydrophobicity). The most recent studies, however, suggest that SOM stability can be interpreted based on bioenergetic constraints experienced by decomposers (Dufour et al. 2022; Henneron et al. 2022; Lehmann et al. 2020).

Stable organic compounds react slowly (Davidson and Janssens 2006) because they must overcome the high energy barrier for decomposition (Rovira et al. 2008; Williams and Plante 2018). Microbial strategies to break down SOM involve the metabolic costs to overcome energetic barriers formed by stabilization mechanisms (Henneron et al. 2022; Rovira et al. 2008). At first glance, this seems related to the adaptationally relevant concept of ‘energy return on investment’ (ROI) (Hall 2017; Harvey et al. 2016; Rovira et al. 2008; Williams and Plante 2018). This impression, however, is inaccurate because the energy investments for e.g., enzyme production, is impossible to relate to the energy return within an unknown number of subsequent biochemical reactions (see details in Box 1) (Dufour et al. 2022).

Because soil microorganisms are strongly limited by energy resources (Gunina and Kuzyakov 2022; Kästner et al. 2021), the SOM pools with lower energy barriers are decomposed first (Rovira et al. 2008). In contrast, organic compounds with the highest *E*_*a*_ are more resistant to microbial decomposition and remain in the soil for a long time. Therefore, we hypothesize that the *E*_*a*_ of organic matter transformation characterizes SOM stability.

Apparently, no systematic comparative studies have examined the activation energies of SOM decomposition (Kästner et al. 2024). Nonetheless, quantifying the *E*_*a*_ of organic matter transformation processes by microbial and chemical pathways is crucial to understand (i) the inherent stability of organic matter and its pools in soil; (ii) the enzymatic decomposition and microbial utilization of organic compounds; (iii) the reaction rates in response to global warming; (iv) the consequences of decoupling carbon and nutrient cycling; and (v) carbon cycle–climate feedback.

This paper generalizes the *E*_*a*_ of specific reactions of SOM transformation by microbial and enzymatic processes, as well as by chemical reactions, clarifies the process rates depending on their mechanisms, and evaluates their response to climate change–soil warming. We also address remaining open questions: (i) What is the difference in energy barrier between enzyme‐catalyzed hydrolysis of organic matter and mineral ions cleavage from organics, as well as complete mineralization of organic matter by microorganisms? (ii) How strongly do enzymes decrease the *E*_*a*_ of organic matter decomposition in soils compared to non‐catalyzed chemical oxidation? (iii) What are the effects of *E*_*a*_ on the transformation of organic compounds in the context of carbon and nutrient cycles under global warming?

## 2 Methods

### 2.1 Database of Activation Energies

We reviewed the activation energies of SOM decomposition and mineralization as well as of enzymatic reactions and thermal oxidation published in peer‐reviewed articles before January 2024. We searched for data using Scopus and Semantic Scholar with the search query ‘activation energy’ AND ‘organic matter’ AND ‘soil’. The following criteria were set up for article selection: (i) only studies on organic matter transformation in soils were included; and (ii) quantitative *E*_*a*_ values and the calculation approach used were clearly described. This yielded 73 papers (Table S1) and 1384 activation energy values (Figure S1). We recorded soil properties (e.g., soil C and N content, pH) when these data were reported. We extracted environmental variables (i.e., geographic: latitude, longitude, elevation; climatic: mean annual precipitation and temperature), ecosystem type (e.g., forest, grassland, cropland), as well as the analytical and calculation approaches used to estimate the activation energy. From the published literature, we collected 911 *E*_*a*_ values determined based on CO_2_ efflux, 253 on potential activities (*V*_*max*_) of various enzymes, 217 on thermal analysis, and 9 based on microcalorimetry. The weightings of the *E*_*a*_ values were not necessary because nearly all included papers used four experimental replicates—this means the *E*_*a*_ values have the same weightings.

To analyze the effects of biochemical processes of organic matter transformation in soils, we grouped the collected data based on analysis methods used to measure and calculate activation energy: oxidative and hydrolytic enzyme activities; CO_2_ production; heat release during soil incubation; thermal oxidation of SOM. The enzyme catalyzed reactions were grouped according to the enzyme groups (hydrolyzing and oxidative enzymes) and element cycles (C, N, P and S).

### 2.2 Approaches to Calculate Activation Energy

Four analytical approaches are commonly used to calculate *E*_*a*_, each characterized by specific mechanisms of organic matter transformation in soil (and each with a broad range of analytical protocols, summarized in Table 1 with more details in Box 2). All four approaches used to calculate the *E*_*a*_ of SOM transformation have specific advantages and limitations and describe specific reaction groups (Table 2).

**TABLE 1:** Overview of analytical approaches and calculation protocols to estimate the activation energy (*E*_*a*_) of SOM transformation.

	Key parameters for analytical protocol	Calculation protocol and references
Thermal analysis	*Techniques for converting SOM to CO*_*2*_ *and other products* – Thermal oxidation (combustion) – Pyrolysis – Pyrolysis and combustion (Rock‐Eval method) *Heating rate* – Single heating rate experiment – Multiple heating rate experiment – 5°C per min… 80°C per min *Heating range* – Up to 600°C–650°C – Up to 1000°C *Metrics* – Mass – Mass change rate – Energy (heat) release	– ‘rampedpyrox’ Python package (Grant et al. 2019; Hemingway et al. 2019; Stoner et al. 2023) – Reich‐Fuoss method (Sokolov et al. 2021) – Coats‐Redfern method (Coats and Redfern 1964; Yan et al. 2020) – Doyle integral method (Yan et al. 2020) – Maximum rate method (Yan et al. 2020) – Vyazovkin method (Gai et al. 2013; Guida et al. 2016; Silva et al. 2019) – DAEM/DAEM simplified method (Ceylan and Kazan 2015; Li et al. 2020; Soria‐Verdugo et al. 2017) – Flynn‐Wall‐Osawa isoconversional method (Li et al. 2020; Thakur et al. 2018) – KAS method (Reinehr et al. 2021; Thakur et al. 2018) – Freeman‐Carroll method (Peng 2001; Rizzo et al. 2013) – Friedman (Guida et al. 2016; Reinehr et al. 2021; Yu et al. 2020) – Starink (Yu et al. 2020) – Popescu (Yu et al. 2020) – FAS (Yu et al. 2020) – FMO (Yu et al. 2020) – Half‐width method (Miranda et al. 2013)
CO_2_ efflux	*Soil incubation temperature steps* – From 2 to 6 *Incubation temperature* – −2°C … +45°C *Temperature increase step* – From 1°C to 20°C *Incubation duration* – From 1 day to several years *Soil moisture* – From 35% to 75% of WHC – Field level *Metrics* – CO_2_ release rate	– Arrhenius equation – *E*_*a*_ is calculated based on slope of ln(*k*) vs. (1/*T*) (Figure 1)
Enzyme activities	*Incubation temperature steps* – From 2 to 9 *Incubation temperature* – 0°C … +70°C *Metrics* – Potential enzyme activity (*V*_*max*_) analytical protocols for enzyme activity: Fluorimetric microplate enzyme assay (Marx et al. 2001); Protocols by Alef and Nannipieri (1995); Protocols by Eivazi and Tabatabai (1988); Protocols by Parham and Deng (2000); and many others	– Arrhenius equation – *E*_*a*_ is calculated based on slope of ln(*k*) vs. (1/*T*) (Figure 1) – Macromolecular Rate Theory (MMRT) (Alster et al. 2016)
Microcalorimetry	*Temperatures for soil incubation* – +15°C … +60°C (potentially up to +100°C) *Duration of the isothermal periods after increasing temperature* – From 15 to 48 h *Heating rate* – 0.017°C–0.083°C per min *Metrics* – Metabolic heat	– Arrhenius equation – *E*_*a*_ is calculated based on slope of ln(*k*) vs. (1/*T*) (Figure 1)

**TABLE 2:** Advantages and limitations of approaches to estimate activation energy of SOM decomposition.

Advantages	Limitations
*Thermal analysis*	
– Analytically simple, rapid, cost‐efficient – Inherent chemical stability of SOM – Independent of the ecophysiological status of microorganisms – Independent of weather and recent environmental effects – Enables assessing the *E*_*a*_ of various SOM pools without their physical separation – Various options for *E*_*a*_ calculation (Box 2)	– Does not reflect the decomposition rates under real soil conditions – Provides solely proxies for biochemical stability – Integrative: does not distinguish between various SOM stabilization mechanisms – Overestimates SOM stability due to the possible formation of stable compounds from labile ones during soil heating
*CO*_*2*_ *efflux*	
– Overall SOM decomposition by all mechanisms under real soil conditions – Analytically simple and cost‐efficient – Process analysis without disturbing the living system – Long‐term incubation enables assessing increasing stability of remaining SOM pools	– Dependent on the ecophysiological status and community structure of microorganisms – Characterizes only a very small portion of easily decomposable SOM pools that are respired (especially in short experiments – less than 2 weeks), and *E*_*a*_ consequently depends on incubation duration – Does not capture energy investments in reactions that do not release CO_2_ as a product (e.g., depolymerization, hydrolysis, sorption/desorption) – Reactions without CO_2_ release are not considered as energy investment to SOM decomposition – Time‐consuming
*Potential enzyme activity*	
– *E*_*a*_ of enzyme‐specific reactions – *E*_*a*_ of reactions without CO_2_ release	– Relates to enzyme activity under substrate saturation (non‐limiting conditions), which is not common in soils – Does not reflect the immediate microbial processes
*Microcalorimetry*	
– Analytically simple, rapid, cost‐efficient – SOM decomposition at simulated natural temperatures and heat release without disturbing the living system – Option to separate bio‐mineralization and direct oxidation of SOM under heat stress conditions (Hansen et al. 2018)	– Low signals especially with low SOM content – Depends on the ecophysiological status and community structure of microorganisms – Characterizes easily decomposable portion of SOM that is respired (especially in short experiments – less than 2 weeks) – Time‐consuming

BOX 2 **Approaches to estimate activation energy (*E*_*a*_) of SOM decomposition.**

**1. Thermal analysis**

“Thermal analysis” is a group of techniques in which a physical or chemical property of a sample is monitored against temperature increase, which is programmed. The most commonly used thermal techniques are Thermogravimetry (which measures weight/mass of a sample as a function of temperature); Differential thermal analysis (technique to measure the difference in temperature between a substance and a reference (often inert) material against temperature); Differential scanning calorimetry (which determines the heat energy release as a function of temperature).

Thermal analysis of soil provides data on the noncatalyzed chemical oxidation of SOM. Soils are controlled‐heated in an (i) oxidizing atmosphere (combustion reaction, SOM oxidation); (ii) inert atmosphere (SOM pyrolysis); or (iii) inert atmosphere with subsequent combustion of pyrolysis products (Rock‐Eval analysis).

Soil is heated to 600°C–650°C (sometimes up to 1000°C) with a single or multiple heating rate from 5°C to 80°C per min. Soil mass loss in the range from ~180°C–200°C to 600°C–650°C corresponds to SOM content.

One‐heating rate methods or multi‐heating rate methods are used to calculate *E*_*a*_.

**FIGURE B1:**
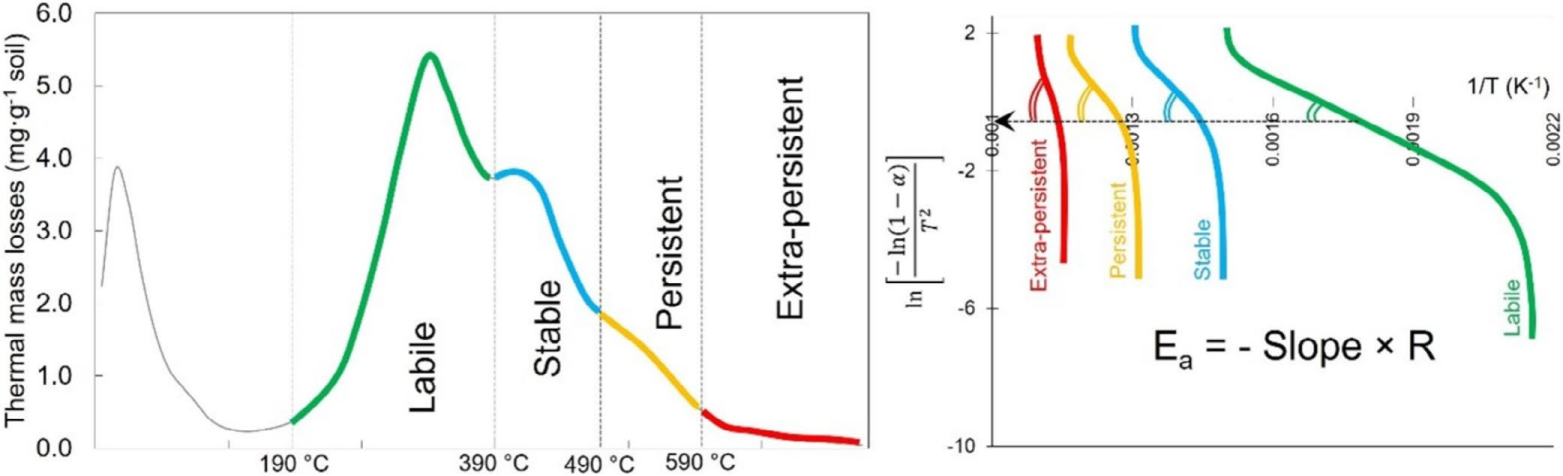
Visualization of the Coats‐Redfern method to calculate the activation energy (*E*_*a*_) of four pools of soil organic matter (SOM): The rate of thermal mass loss during soil heating at one rate is used. SOM combustion is considered as a multistage reaction. *E*_*a*_ is calculated for each stage (here four stages) separately as the slopes of the Arrhenius plot of the natural logarithm of reaction rates vs. (1/*T*) (please see Figure 2). The increase in the curve slope from the thermally labile SOM (green line) to the thermally persistent SOM (red line) corresponds to a rise in their *E*_*a*_.

**1.1. One‐heating rate methods** (e.g., Coats‐Redfern method):

The rate of thermal mass loss during soil heating at one rate is used. SOM combustion is considered as a multistage reaction. *E*_*a*_ is calculated for each stage separately as the slopes of the Arrhenius plot of the natural logarithm of reaction rates vs. (1/*T*) (Figure B1). The increase in the curve slope from the thermally labile SOM (green line) to the thermally persistent SOM (red line) corresponds to a rise in their *E*_*a*_.

**FIGURE B2:**
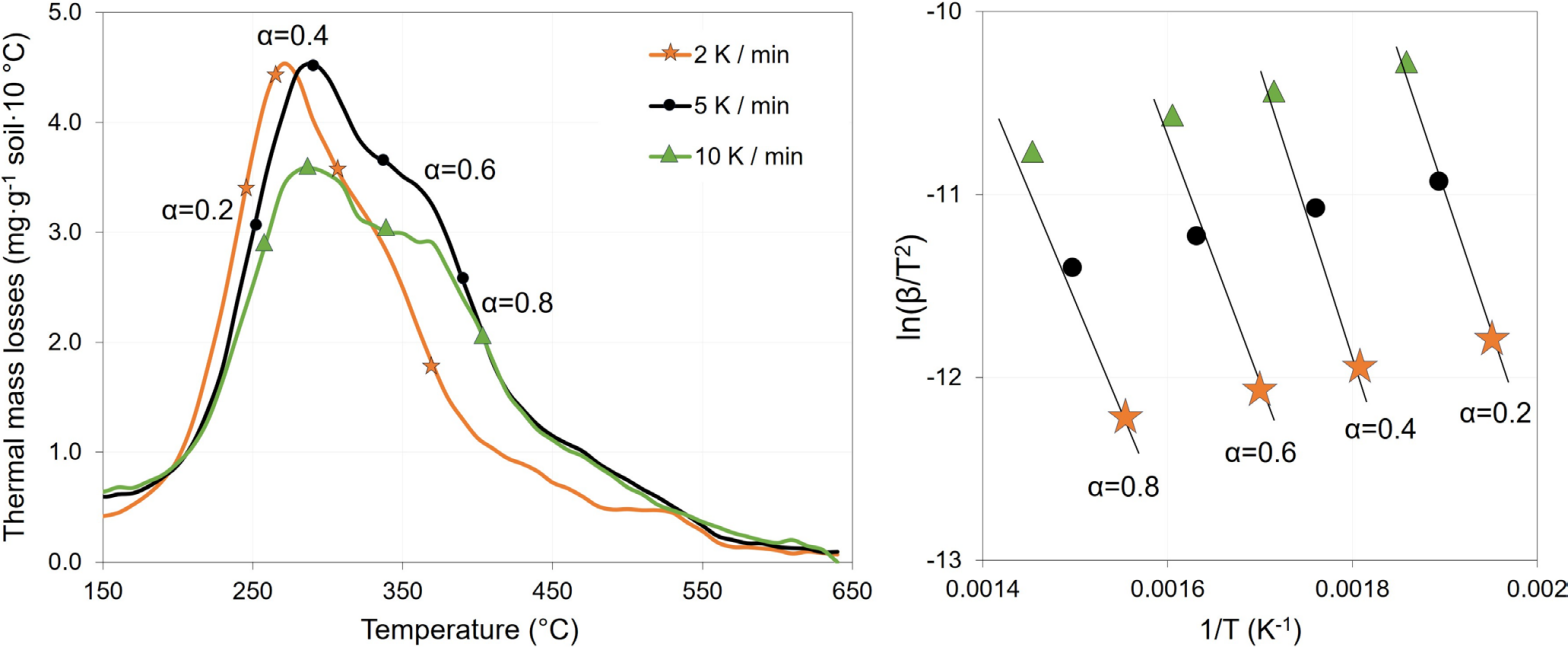
Visualization of the Friedman method to calculate the activation energy (*E*_*a*_) of soil organic matter (SOM) decomposition under increasing temperature: The soil is heated at several rates, usually from 3 to 5 rates (here exemplary at 3 rates). The differential thermal curves are used to find the points corresponding to e.g., 20%, 40%, 60%, and 80% of SOM loss for each heating rate, which are graphically transferred to an Arrhenius plot for *E*_*a*_ calculation as slopes of the natural logarithm of the ratio of the heating rate and temperature squared (ln(*β*/*T* ^2^)) vs. (1/*T*).


**1.2. Multi‐heating rate methods** (e.g., Friedman method):

The soil is heated at several rates, usually from 3 to 5 rates (Figure B2). The differential thermal curves are used to find the points corresponding to e.g., 10% … 90% SOM loss for each heating rate, which are graphically transferred to an Arrhenius plot for *E*_*a*_ calculation as slopes of the natural logarithm of the ratio of the heating rate and temperature squared (ln(*β*/*T* ^2^)) vs. (1/*T*).

The activation energy analysis should be combined with the pre‐exponential factor and reaction model (Šimon et al., 2022) for a better understanding of the SOM transformation.

**2. CO**_**2**_ **efflux**

**FIGURE 2:**
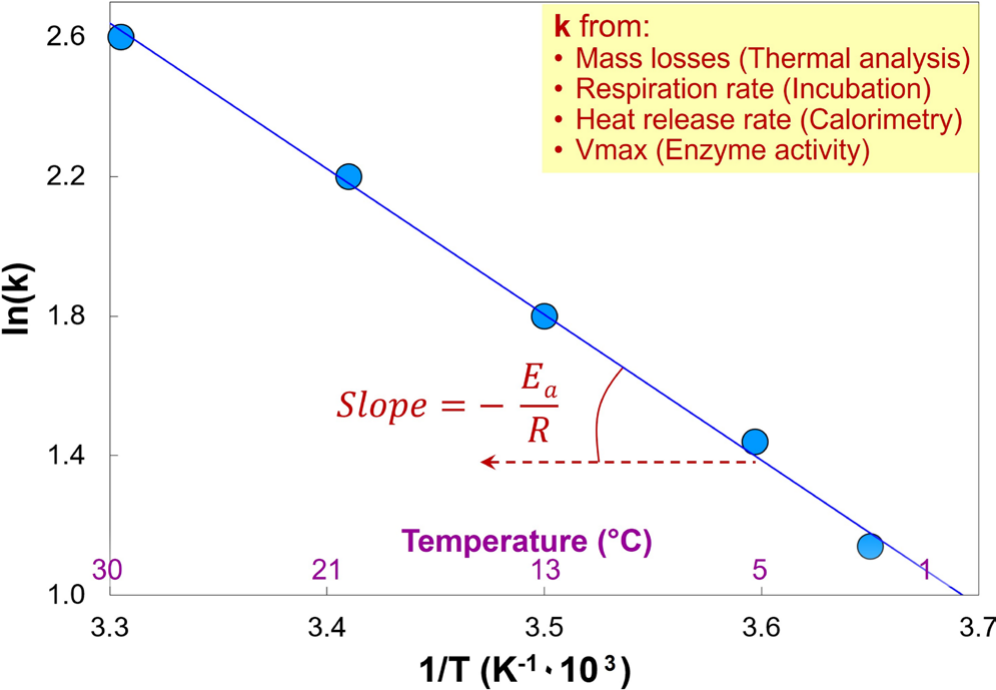
Visualization of the principle of the activation energy (*E*_*a*_) calculation based on the Arrhenius equation. *E*_*a*_ is calculated by multiplying the gas constant (R) by the slope of the natural logarithm of the reaction rate (*k*) vs. the inverse absolute temperature of reaction (*T*). The temperature in °C is presented in violet on the X axis, corresponding to the 1/*T* values in Kelvin^−1^.

This approach is based on soil incubation at a temperature gradient and on measurements of rates of microbially respired CO_2_. Rates of CO_2_ efflux as a function of soil temperature are used to create an Arrhenius plot of microbial respiration rates vs. (1/*T*) (Figure 2). *E*_*a*_ of microbial SOM decomposition is calculated by the slope of this plot.

**3. Potential enzyme activity**

This approach is based on measurements of potential enzyme activity (*V*_*max*_) at a temperature gradient. The *E*_*a*_ of individual enzymatic reactions or the enzyme‐catalyzed hydrolysis leading to SOM decomposition is calculated from an Arrhenius plot by the slope of reaction rates vs. (1/*T*) (Figure B1).

**4. Microcalorimetry**

Microcalorimetry is used to determine the specific heat released from physical, chemical, and biological processes (here related only to processes in soil). The reaction enthalpy is obtained by dividing the reaction heat by the amount of the consumed substrate and considering a potential pressure rise due to respiration. The pressure correction is typically very small. The *E*_*a*_ of SOM decomposition is calculated from the Arrhenius plot by the slope of the ratio between the rate of heat release and (1/*T*).

Calorimetric measurements of heat release rates from soil incubated at various temperatures are the basis to calculate the *E*_*a*_ of microbial decomposition of organic matter. Rates of CO_2_ release during soil incubation as a function of temperature are used to estimate the *E*_*a*_ of microbial mineralization of organic matter. The *E*_*a*_ of enzyme‐catalyzed hydrolysis of polymers and cleavage of mineral ions (phosphate, ammonium and sulphate) is calculated based on the potential activity (*V*_*max*_) of enzymes depending on temperature. Calculating the *E*_*a*_ of noncatalyzed chemical mineralization of SOM involves heating the soil up to extremely high temperatures (600°C–650°C and higher) in an oxidizing (synthetic air or laboratory air) or inert (N_2_ or He) atmosphere, combined with measuring the rate of organic matter losses during combustion or pyrolysis.

All approaches used to calculate the *E*_*a*_ of SOM transformation are based on the Arrhenius equation (Box 1), regardless of the conversion mechanisms of SOM to CO_2_ and other products:

(1) $$E_{a}=-R\times T\times \ln\left(\frac{k}{A}\right)$$

where *k* is the reaction rate, *T* the absolute temperature in Kelvin, *R* the gas constant, and *A* the pre‐exponential factor.

In practice, *E*_*a*_ is defined as the slope of the natural logarithm of the reaction rate vs. the inverse absolute temperature (Figure 2).

## 3 Results and Discussion

### 3.1 Activation Energy of Soil Organic Matter Transformations

**FIGURE 3:**
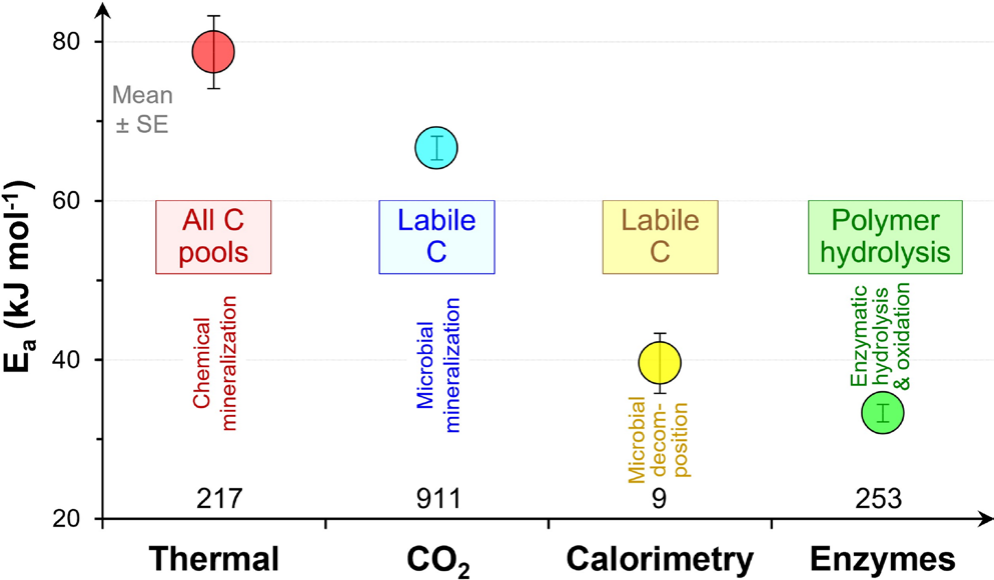
Means and standard errors for the activation energy (*E*_*a*_) of four groups of SOM transformation processes: (i) chemical oxidation (via thermal analysis as pure chemical oxidation of total SOM); (ii) microbial mineralization (analyzed by CO_2_ efflux from soil); (iii) microbial decomposition (analyzed by heat release from soil); (iv) enzyme‐catalyzed polymers hydrolysis and mineral ions cleavage (analyzed by potential enzyme activity) (see details in Table 1 and Box 2). X axis: The number of *E*_*a*_ values for each method of calculations. The distribution of *E*_*a*_ values for each approach is available in Supporting Information (Figure S1).

The mean activation energies of SOM transformation processes were ranked as follows: *E*_*a*_ of chemical oxidation > *E*_*a*_ of microbial mineralization > *E*_*a*_ of microbial decomposition > *E*_*a*_ of enzyme‐catalyzed hydrolysis of polymers and cleavage of mineral ions (Figure 3).

### 3.2 Activation Energy of Chemical Oxidation of Soil Organic Matter

The mean *E*_*a*_ of chemical oxidation of SOM is 79 ± 5 kJ mol^−1^ (Figure 3). This value reflects the energy barrier to combust the whole SOM to CO_2_, H_2_O, and other oxides, and thus reflects the internal chemical stability (inherent stability) of SOM. Combustion reactions of SOM to CO_2_ in an oxidizing atmosphere have a 2.1‐times lower *E*_*a*_ than that of SOM pyrolysis under inert atmosphere (Figure S2). Accordingly, reactions with oxygen reduce the *E*_*a*_ to pyrolysis due to the strong electron acceptor potential of O_2_. The use of only internal electron acceptor resources, as in SOM pyrolysis, increases the energetic barrier of the reaction and enormously reduces its rate. This directly explains the accumulation (absence of decomposition) of organic matter under anoxic conditions (e.g., peats, aquatic sediments) and the much slower SOM decomposition in paddies compared to upland soils (Wei et al. 2021).

The resistance of SOM to thermal oxidation was attributed to its chemical stability (Rovira et al. 2008) and to soil texture (Stoner et al. 2023). Soil minerals, especially clays and Fe and Al (oxyhydr)oxides, strongly bind organic molecules (Feng and Simpson 2008; Plante et al. 2011; Rovira et al. 2024; Stoner et al. 2023). The *E*_*a*_ of chemical mineralization of SOM is not affected by soil moisture, temperature, pH, enzymatic activity, functional diversity of microorganisms, or their seasonal and diurnal dynamics (An et al. 2023; Leifeld and von Lützow 2014). This makes SOM thermal stability the most valid measure to compare the inherent organic matter stability across soil types and depths (An et al. 2023; Plante et al. 2009, 2011) as well as land uses (Filimonenko, Kurganova, et al. 2024). We therefore suggest that the *E*_*a*_ of chemical oxidation (e.g., analyzed by thermal analysis) is the best measure of inherent SOM stability.

The structural and molecular heterogeneity of SOM, combined with a variety of mechanisms of its physical and chemical stabilization in soil (von Lützow et al. 2006), determines the differences in the *E*_*a*_ of the chemical mineralization of individual SOM pools. The value for thermally labile SOM (combusted below 400°C) is lower than that of thermally stable pools (> 400°C) (Filimonenko, Kurganova, et al. 2024; Sanderman and Grandy 2020; Williams et al. 2014). Even though this may be affected by the correlation between *E*_*a*_ and combustion temperature, which limits interpretability (Vyazovkin 2025), the thermally stable SOM is more resistant to breakdown. The breakdown of stable SOM requires more energy inputs and therefore remains undegraded longer in soil than the pools with low thermal stability (Plante et al. 2011; Williams and Plante 2018).

SOM is a mixture of pools with various chemical persistence and thermal stability. Lignin (a precursor of SOM) can be partly chemically oxidized under soil conditions, unlike cellulose and hemicellulose, which are mainly enzymatically decomposed (Kögel‐Knabner 2002). Lignin decomposition involves (per)oxidative enzymes such as laccases, manganese peroxidase, lignin peroxidase, and versatile peroxidases. In contrast to enzymatic decomposition, the chemical oxidation of lignin can occur spontaneously (Hansen et al. 2018; Yu and Kuzyakov 2021). The role of such abiotic lignin oxidation for its total mineralization increases under conditions unfavorable for microbial activity, e.g., dry and hot deserts, high UV radiation. The greater contribution of lignin to the CO_2_ flux by chemical mineralization of SOM (Hansen et al. 2018) reflects its lower *E*_*a*_ compared to carbohydrates (Markou et al. 2012) (Figure S3).

### 3.3 Activation Energy of Microbial Decomposition and Mineralization of Soil Organic Matter

Microbial decomposition of soil organic matter precedes its complete mineralization. The two processes have specific *E*_*a*_ and are distinguished here (Box 1): *SOM decomposition* is the sum of chemical breakdown and all biochemical transformations of organic materials yielding simple organic and inorganic molecules. *SOM mineralization* is the result of SOM transformation to CO_2_ and H_2_O and other inorganic molecules, indicating the complete oxidation of organic matter. Most studies do not clearly differ between the two because useful methods to separate and measure them in situ are absent. The first process, however, is identified by the heat flux generated by a series of hydrolytic and oxidative enzymatic reactions, and the second—by the CO_2_ flux (and e.g., N mineralization in other studies).

#### 3.3.1 Activation Energy of Microbial Mineralization of Organic Matter in Soil

The microbially‐driven SOM mineralization (complete decomposition to CO_2_, H_2_O, NH_4_^+^ etc.) has an *E*_*a*_ of 67 ± 1 kJ mol^−1^ (Figure 3). The *E*_*a*_ calculation of SOM mineralization to CO_2_ depending on temperature is widely used (Craine et al. 2010; Lefèvre et al. 2014; Wagai et al. 2013), but has three specific limitations: (i) *E*_*a*_ depends on the ecophysiological status (activity) and community structure of microorganisms (An et al. 2023), producing various isoenzymes that differently lower *E*_*a*_ for the same reaction (Razavi et al. 2016); this is affected by land‐use type, ecosystem type, edaphic factors, and fresh input of available organics (Craine et al. 2010; Leifeld and von Lützow, 2014); (ii) *E*_*a*_ for CO_2_ production characterizes only easily decomposable SOM pools, which are respired (within the duration of incubation experiment), and provides no information about *E*_*a*_ of total SOM; (iii) *E*_*a*_ does not consider many SOM transformation reactions that do not produce CO_2_ (e.g., depolymerization, hydrolysis reactions, cleavage of mineral ions, sorption/desorption) (Barros et al. 2015, 2016).

**FIGURE 4:**
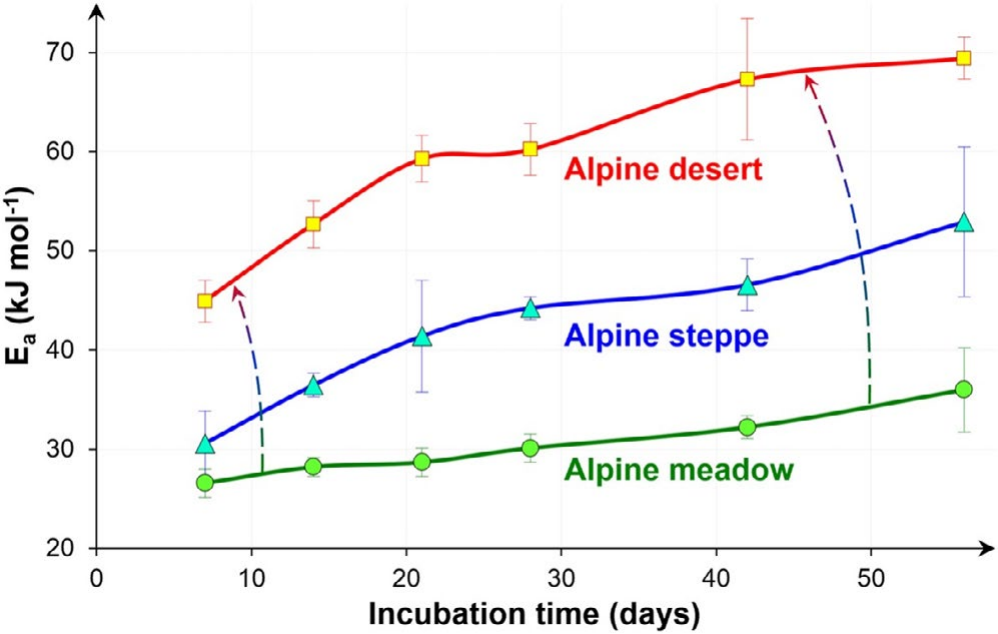
Effects of soil incubation duration on activation energy (*E*_*a*_ ± standard error) of microbial mineralization of soil organic matter. Soil from three main grassland types on the Qinghai‐Tibet Plateau: Alpine meadow, Alpine steppe, Alpine desert (Li et al. 2015, modified). Dashed arrows show the increase of the differences in the *E*_*a*_ between the three soils due to depletion of labile organic matter during incubation.

Microorganisms preferentially utilize easily available substrates (Dufour et al. 2022; Gunina and Kuzyakov 2022; Harvey et al. 2012; Rovira et al. 2008). Accordingly, only the most biogeochemically labile SOM pools with the lowest *E*_*a*_ are utilized over short‐term incubations. This means that *E*_*a*_ of the microbial mineralization increases with the soil incubation time (Barros et al. 2016; Li et al. 2015) (Figure 4). The depletion of biochemically labile SOM pools and prolonged microbial utilization of SOM requires overcoming the higher *E*_*a*_ for the remaining more stable pools (Hartley and Ineson 2008; Henneron et al. 2022). The higher value of those pools explains a slower decomposition rate with incubation duration (Bosatta and Ågren 1999). These results of short incubation experiments (even months or years) regarding slower decomposition over time are confirmed by long‐term (25–80 years) bare fallow experiments under field conditions (Barré et al. 2010; Lefèvre et al. 2014).

This raises an important question: Can the *E*_*a*_ of chemical mineralization of SOM be directly used to assess microbial mineralization of organic matter under soil conditions? The values in a set of 19 grassland soils in Switzerland (Leifeld and von Lützow 2014) were analyzed by two approaches: (i) CO_2_ efflux from soil, and (ii) thermal analysis. Comparing the *E*_*a*_ of the two methodologies for the same soil samples helps assess the susceptibility of organic matter to chemical and microbial mineralization (Leifeld and von Lützow 2014; Peltre et al. 2013). The *E*_*a*_ of the former was ~1.6 times higher than that of the latter, and there was no correlation between them (Leifeld and von Lützow 2014). Consequently, the value for chemical mineralization of the whole continuum of SOM compounds is not quantitatively applicable to assess the microbially driven mineralization of the easily available SOM pools (An et al. 2023; Kästner et al. 2024; Leifeld and von Lützow 2014). We speculate, however, that the absent correlation reflects the incomparable pools of SOM mineralized by microbial and chemical pathways. Only a few percent of soil organic C are respired as CO_2_ during microbially driven mineralization compared to 100% release at~600°C to 6500°C or higher. The small percentage of respired CO_2_ during a few days or weeks of incubation reflected solely the most easily available and decomposable SOM pool. The suggestion that the thermally labile pool is the most easily decomposed by microorganisms has been widely postulated (Doležalová‐Weissmannová et al. 2023; Sanderman and Grandy 2020; Williams et al. 2014). Only a few studies, however, confirmed this based on a decrease in the labile SOM content after long‐term (~2 years) soil incubations (Plante et al. 2011) or on a larger cumulative CO_2_ efflux from soil with lower thermal stability (An et al. 2023).

#### 3.3.2 Activation Energy of Microbial Decomposition of Organic Matter in Soil

The *E*_*a*_ of microbially‐driven SOM decomposition is 40 ± 4 kJ mol^−1^ (Figure 3). That process produces both CO_2_ and heat (Endress, Chen, et al. 2024). The flows of respired CO_2_ and released heat, however, are not synchronous because (i) some processes occur with heat release but without CO_2_ production: hydrolysis of macromolecules and polymers, sorption/desorption etc. (Wang and Kuzyakov 2023); (ii) there is a time‐lag between processes of energy gain (with CO_2_ release) and storage in e.g., ATP, whereby this energy will be used and released later (without CO_2_ production), e.g., protein synthesis. This raises a key question about the necessity of exactly synchronizing the analytical equipment for the dynamics of heat release (microcalorimetry) with that for CO_2_ measurements (e.g., IRGA), whereby the cumulative heat and CO_2_ release should be balanced (Maskow et al. 2024).

Assessing the intensity of microbial SOM decomposition is based on the heat release (and analysis e.g., by calorimetry) from all stages of organic matter transformation (Critter et al. 2001; Herrmann et al. 2014). Microcalorimetry captures the released heat as the thermodynamic balance resulting from the full range of processes: the exothermic and endothermic chemical and microbial reactions (Kästner et al. 2024; Rong et al. 2007). Considering the large difference of the *E*_*a*_ values for heat release and CO_2_ production (Figure 3), the responsible processes strongly differ. This questions the meaning and applicability of the calorespirometric ratio, frequently used to assess microbial metabolic fluxes in soil (Barros et al. 2015; Yang et al. 2024).

### 3.4 Activation Energy of Enzyme‐Catalyzed Polymers Hydrolysis and Mineral Ions Cleavage

The mean *E*_*a*_ of enzyme‐catalyzed hydrolysis of organic polymers and cleavage of mineral ions in SOM is 33 ± 1 kJ mol^−1^ (Figure 3). Microbial decomposition of high molecular organic compounds includes many parallel and sequential series of enzyme‐catalyzed reactions with specific *E*_*a*_ (Das et al. 2024; Nannipieri 2020; Nannipieri et al. 2025). These catalytic effects reduced the *E*_*a*_ of chemical mineralization by 46 kJ mol^−1^ (Figure 3). Assuming the equivalence of the *A* coefficient from the Arrhenius equation for noncatalyzed chemical oxidation and enzyme‐catalyzed SOM decomposition, as used by Leifeld and von Lützow (2014), the potential acceleration of enzymatic transformation compared to chemical processes is ~10^8^‐fold. This acceleration is enormous and describes the *potential* effects of enzymes (*V*_*max*_) when (i) the available substrates are not limited, (ii) the reactions proceed in solution, and (iii) organic compounds and enzymes are not protected by physical mechanisms (e.g., entrapping in microaggregates and nanopores) (Blagodatskaya et al. 2016; Nannipieri et al. 2018). These conditions are rare in soils. This calls for caution regarding the previous suggestion that the higher CO_2_ production from soil due to the catalytic effects of enzymes is 1.5 × 10^7^‐fold compared to its release under the noncatalyzed chemical reactions (Leifeld and von Lützow 2014).

The efficiency of catalytic reactions under real soil conditions is limited (Feng and Simpson 2008) by: (i) unsaturation of organic substrates, (ii) spatial heterogeneity of organic compounds (Endress, Dehghani, et al. 2024), (iii) mismatch in the localization of substrates and enzymes (Bilyera et al. 2021; Zhang et al. 2023; Kravchenko et al. 2019), (iv) enzyme immobilization on soil minerals (Sarkar et al. 1989; Wu et al. 2014; Zimmerman and Ahn 2010), and (v) partial enzyme deactivation (Wang and Kuzyakov 2023). Based on the 12 kJ mol^−1^ difference between the *E*_*a*_ of chemical and microbial processes (Figure 3), the catalytic acceleration of CO_2_ efflux from soil is 140 times (at 20°C). This huge gap between the potential (~10^8^‐fold, thermal oxidations vs. enzyme‐catalyzed decomposition) and actual effects (140 times, thermal vs. microbial based on CO_2_ efflux) of enzymes in accelerating SOM transformation partly explains why organic matter remains undecomposed in soils for very long periods compared to its utilization in solution. We assess that every 1 kJ mol^−1^ decrease in *E*_*a*_ due to the catalytic effect of enzymes corresponds to a 1.5‐times (+50%) increase in the SOM decomposition rate under soil conditions.

**FIGURE 5:**
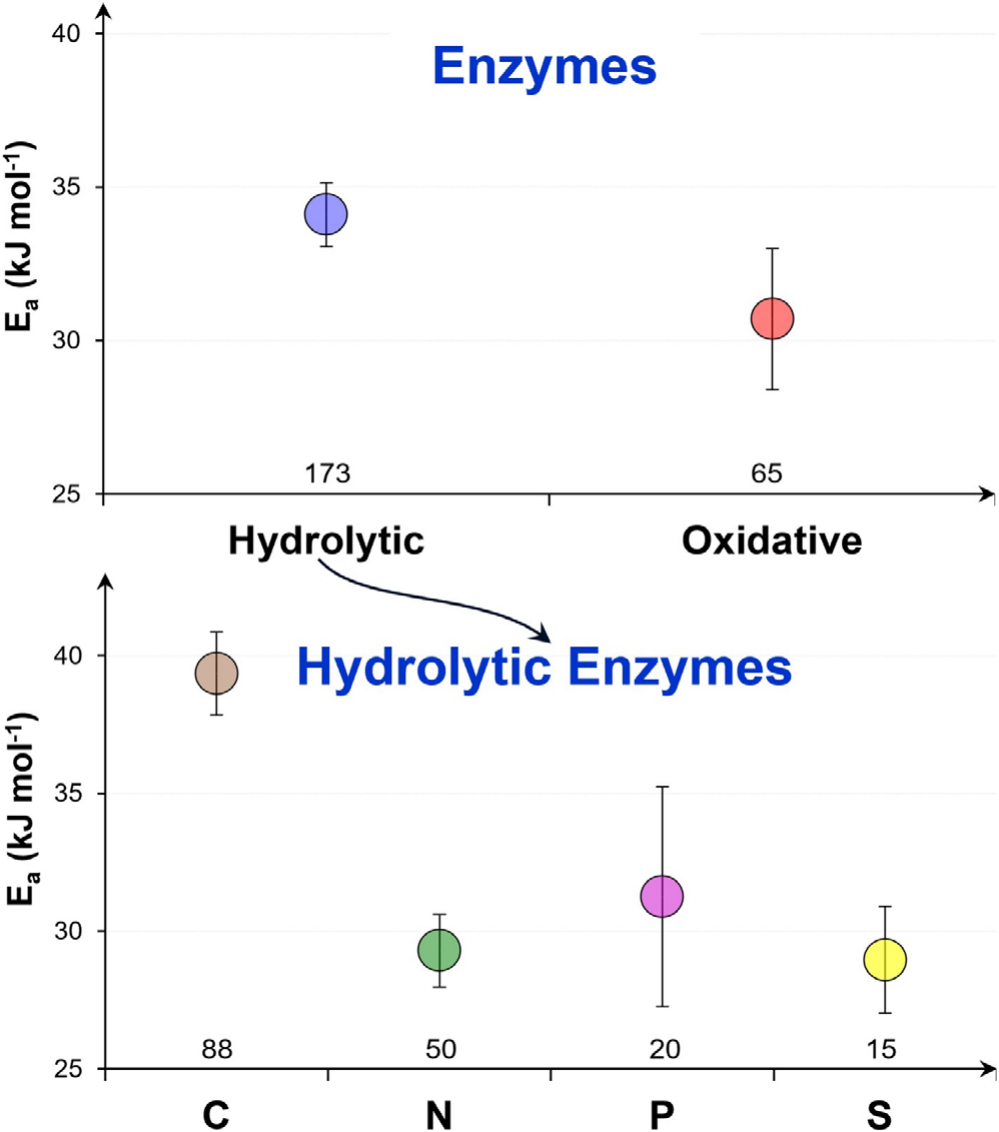
Activation energy (*E*_*a*_) of hydrolytic and oxidative enzymatic reactions of soil organic matter (SOM) decomposition and *E*_*a*_ of hydrolytic enzymes of carbon (C), nitrogen (N), phosphorus (P), and sulfur (S) cycles. Means and standard errors are presented. X axis: The number of *E*_*a*_ values. The *E*_*a*_ for N‐containing compounds was calculated based on *E*_*a*_ for β‐glucosaminidase, N‐acetyl‐glucosaminidase, leucine aminopeptidase, urease, arylamidase, BAA‐protease, casein‐protease, L‐asparagine amidohydrolase, and tyrosine aminopeptidase. The *E*_*a*_ for P‐containing compounds was based on *E*_*a*_ for acid and alkaline phosphatases and pyrophosphatase; the *E*_*a*_ for S‐containing compounds was based on *E*_*a*_ for arylsulfatase; the *E*_*a*_ of hydrolysis of organic compounds (without N, P, and S) based on β‐glucosidase, β‐D‐fucosidase, cellulase, cellobiohydrolase, and xylanase; the *E*_*a*_ of oxidative enzyme reactions was based on *E*_*a*_ for peroxidase, lignin peroxidase, and phenol oxidase.

SOM decomposition starts with enzyme‐catalyzed reactions hydrolyzing polymers and N‐, P‐, and S‐containing organic compounds. Hydrolases are substrate‐specific: their structure enables catalyzing reactions that cleave specific bonds in organic matter. The oxidation of hydrolyzed and non‐hydrolyzed organic compounds is ongoing by oxidative enzymes. Oxidases use molecular oxygen, hydrogen peroxide, or other reactive oxygen species to oxidize a broad range of molecules that share similar bonds (Paul and Frey 2024; Zavarzina et al. 2025). The energy barrier for specific decomposition reactions (catalyzed by hydrolases) is higher than for non‐specific oxidative processes (catalyzed by oxidases) (Figure 5). The *E*_*a*_ of enzymatic reactions hydrolyzing cleavage of N, P, and S from organic compounds is 9 kJ mol^−1^ lower than that of enzymatic hydrolytic depolymerization of other (N‐, P‐ and S‐free) organic substances (Figure 5). The oxidative reactions for all organic compounds have the same rates as hydrolysis reactions for organic substances containing N, P, or S. Consequently, the turnover of organics containing these elements in soil is much faster than the cycling of N‐, P‐, or S‐free organic compounds (Soldatova et al. 2024).

**FIGURE 6:**
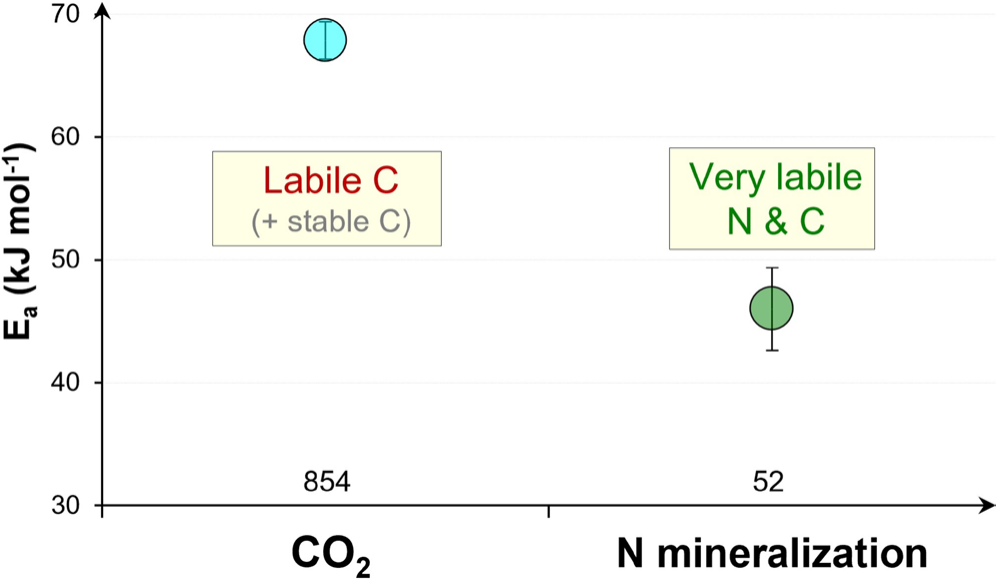
Activation energy (*E*_*a*_) of mineralization of soil organic C and N. Means and standard errors are presented. X axis: The number of *E*_*a*_ values.

Lower‐quality SOM (high C/N ratio) requires a higher *E*_*a*_ (Bosatta and Ågren 1999; Quan et al. 2014). The lower *E*_*a*_ of N mineralization (e.g., hydrolytic cleavage of NH_4_^+^ from amino acids) vs. organic C mineralization (Figure 6) is the result of N mineralization in the most biochemically labile SOM (low C/N ratio, e.g., proteins, amino sugars), which is the main source of available N for microorganisms. The *E*_*a*_ of N mineralization presented in the literature reflects only the N *net*‐mineralization, which, however, is the result of both N gross‐mineralization and N immobilization. Because nearly all these *E*_*a*_ values are obtained from incubation experiments without the addition of organic C, the mineral N content (NH_4_^+^ and NO_3_^−^) increases in soil continuously. Consequently, mineral N is in excess of remaining available C because the already decomposed available C will be lost as CO_2_. This means that N immobilization processes can be neglected because available C (mainly lost as CO_2_) is absent, and that the provided *E*_*a*_ values (Figure 6) therefore correspond to N gross‐mineralization.

Mineral‐associated organic matter (MAOM) contains most of the soil organic N (Georgiou et al. 2025; Stoner et al. 2023). Because of the high physico‐chemical stability of MAOM, N from this slow pool of SOM has a high *E*_*a*_ of mineralization and can hardly be decomposed by microorganisms. This limits N availability in most natural ecosystems (LeBauer and Treseder 2008; Menge et al. 2012).

## 4 Consequences for Soil Organic Matter Decomposition Under Global Warming

Activation energy is a key factor defining the rate of chemical reactions—the threshold between the average energy of molecules at standard temperature and the energy required for the reaction. As the temperature increases, the threshold for the energy barrier decreases (Figure 1) (Menzinger and Wolfgang 1969). The different mean *E*_*a*_ values of various organic matter transformations in soil (Figure 3) suggest that the respective processes will be affected under global warming. This is because the reactions with high *E*_*a*_ are more strongly accelerated by temperature increases than reactions with low *E*_*a*_ (Davidson and Janssens 2006). Considering the *E*_*a*_ of the processes evaluated above, we describe below how global warming will change the cycles of C and nutrients by biotic and abiotic processes of SOM decomposition.

### 4.1 Nutrient Cycles

Global warming affects cycles of C and nutrients (N, P, S) in soil through a set of mechanisms including: (i) changed input amounts and quality of litter and rhizodeposits by plants, (ii) microbial community shifts and subsequent qualitative and quantitative changes in enzyme systems (Cheng et al. 2017; Razavi et al. 2017); (iii) increased solubility of some organic compounds; and (iv) increased decoupling of decomposition of organic compounds with different internal stabilities, i.e., activation energies of their decomposition. Here, we focus solely on the consequences of warming related to the last group of mechanisms directly connected with the differences in *E*_*a*_ of organic matter pools.

The *E*_*a*_ of specific reactions involved in SOM transformation processes is crucial to better understand the effects of global warming on C, N, P, and S cycles. The decomposition of substances with higher *E*_*a*_ is more sensitive to temperature increase compared to compounds with low *E*_*a*_ (Conant et al. 2008; Craine et al. 2010). Considering the 30% higher *E*_*a*_ of hydrolysis of N‐, P‐, and S‐free organic compounds compared to cleavage of NH_4_^+^, HPO_4_^2−^ and SO_4_^2−^ from the organic compounds with these nutrients (Figure 5), global warming will accelerate soil organic C cycling much more than that of N, P, or S.

The higher *E*_*a*_ of stable SOM pools also reflects their stronger response to warming compared to labile SOM pools. Accordingly, stable organic C will be intensively involved in biological C cycling, leading to shorter mean residence times of C in soil. This fully agrees with many studies based on other experimental and analytical approaches showing that global warming will destabilize the persistent SOM pools (Henneron et al. 2022). The C/N ratio of stable pools (e.g., mineral associated organic matter, MAOM), however, is much lower than that of plant residues and particulate organic matter (POM) (Chang et al. 2024). Consequently, opposite to the conclusion based on enzymatic hydrolysis of N, P, and S free organic compounds (see above), nutrients will be involved in cycling in soil more than C.

### 4.2 Abiotic Mineralization of Organic Matter in Soil Under Warming

Corresponding to the high *E*_*a*_ of chemical (abiotic) mineralization of SOM, the oxidation rates by atmospheric oxygen in soil are extremely slow at ambient temperatures (Hansen et al. 2018; Leifeld and von Lützow 2014). Even though the contribution of direct chemical oxidation is very low compared with microbial mineralization (Kögel‐Knabner 2002; Maire et al. 2013), the noncatalyzed abiotic processes are relevant for the C cycle under extreme environmental conditions such as heat stresses or droughts, high UV radiation, or changing redox conditions along with the production of reactive oxygen species (Hansen et al. 2018; Yu and Kuzyakov 2021).

The rates of enzyme‐catalyzed hydrolysis of SOM decomposition increase with temperature only up to 30°C–50°C (Hansen et al. 2018). Temperatures above 50°C rapidly reduce or completely stop the activities of β‐glucosidase, α‐1,4‐glucosidase, cellobiohydrolase, N‐acetyl‐glucosaminidase, phosphatase, and sulfatase because of enzyme denaturation (Riah‐Anglet et al. 2015; Waldrop and Firestone 2004). This reduces enzymatic decomposition under frequent heat stress (Conant et al. 2011), which is already common in many topsoils and expected to increase under further climate warming and wildfires (Filimonenko, Uporova, et al. 2024; Pei et al. 2023; Zhou et al. 2023). Considering the above, the stability of organic matter remaining after decomposition of labile SOM pools will rapidly increase (Figure 4), as will the initial differences between soils.

Parallel to this, extremely high soil temperatures, e.g., common under heat waves, increase the rate of chemical SOM mineralization (e.g., by reactive oxygen species). This is because this reaction has a much higher *E*_*a*_ compared to the respective enzyme‐catalyzed processes (Figure 3) and is therefore most affected by warming. Based on the *E*_*a*_ of chemical mineralization (79 kJ mol^−1^; Figure 3), warming from 20°C to 21°C will increase the rate of direct chemical oxidation by 14%, and a temperature increase to 22°C would boost this rate by 32%. This example, however, does not consider extreme high soil temperatures: without dense vegetation and direct sunshine, the temperature of the soil surface frequently increases up to more than 70°C. Under such conditions, nearly all enzymatic reactions cease, whereas abiotic decomposition of organic matter increases.

The abiotic mineralization (here, incineration) of organic matter is common in areas with very intensive UV radiation and sparse vegetation, e.g., Atacama Desert (Figure S4), high altitude areas, all equatorial deserts, Australian Outback, and polar regions. Considering the increasing intensity of UV radiation, the incineration of all organics on the soil surface will rise. We assume that the relative importance of this abiotic decomposition of soil surface organic matter will rise because high soil temperatures will stop enzymatic and microbial decomposition.

## 5 Conclusions

**FIGURE 7:**
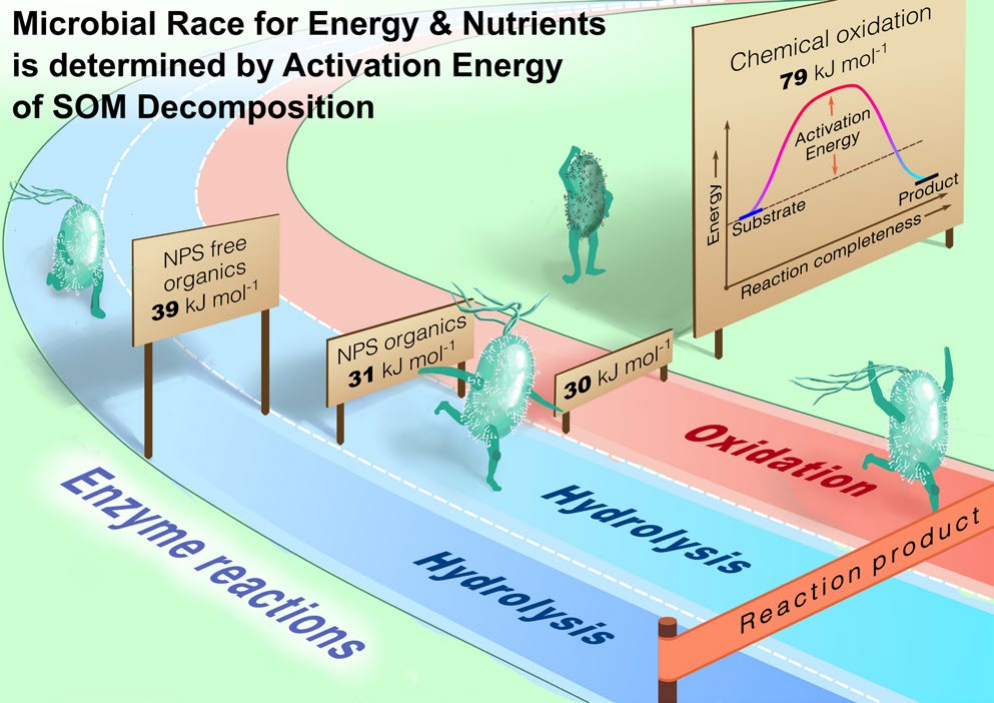
‘Microbial race’ for energy and nutrients (N, P, S) as determined by activation energy (*E*_*a*_) of soil organic matter (SOM) transformations by enzyme‐catalyzed reactions. The increase in *E*_*a*_ is accompanied by a decrease in the reaction rate. The higher *E*_*a*_ of enzyme‐catalyzed hydrolysis of C than of N, P, and S reflects faster acceleration of the C cycle compared to nutrient cycling, leading to their decoupling by global warming. The *E*_*a*_ values of the enzyme‐catalyzed reactions (as well as of the chemical oxidation) correspond to those in Figures 3 and 5.

This review raises the importance of the fundamental parameter of (bio)chemical reactions—the activation energy (*E*_*a*_) – and generalizes it for processes of organic matter transformation in soil. Based on the generalization of *E*_*a*_ values published in the literature as assessed by four approaches (thermal oxidation of SOM, CO_2_ efflux by soil incubation, enzyme activities, and heat release measured by microcalorimetry), we show the principal differences between the rates of chemical and microbial mineralization of SOM, as well as of enzyme‐catalyzed processes of C, N, P, and S cycles (Figure 7).

The *E*_*a*_ of chemical mineralization of SOM (thermal oxidation) was 16% higher than that of microbial mineralization to CO_2_, corresponding to 140 times faster microbially (enzyme) driven reactions. Every 1 kJ mol^−1^ decrease in *E*_*a*_ due to the catalytic effect of enzymes corresponds to a 1.5 times (+50%) increase in the SOM oxidation rate under soil conditions. During continuous microbial decomposition of SOM, the *E*_*a*_ increases because the preferably utilized available pools with lower *E*_*a*_ will be exhausted, and the *E*_*a*_ of the remaining stable pools is (much) higher. The 30% higher *E*_*a*_ value of enzyme‐catalyzed hydrolysis of N‐, P‐, or S‐free organic compounds than of the organics containing those elements reflects the faster acceleration of the C cycle with increasing temperatures compared to nutrient cycling. We therefore expect that global warming will decouple C from nutrients and more strongly limit C for microorganisms in soil.

In conclusion, the *E*_*a*_ of organic matter transformation processes in soil is simple to determine by one of the four approaches or their combination. Importantly, *E*_*a*_ reflects the rates of the main groups of (bio)chemical reactions as well as the intrinsic stability of organic matter in soil. It is therefore very useful to predict the acceleration of the SOM transformation by global warming.

## Author Contributions

**Ekaterina Filimonenko:** conceptualization, data curation, formal analysis, funding acquisition, investigation, methodology, validation, visualization, writing – original draft, writing – review and editing. **Yakov Kuzyakov:** conceptualization, formal analysis, funding acquisition, investigation, methodology, project administration, supervision, visualization, writing – original draft, writing – review and editing.

## Conflicts of Interest

The authors declare no conflicts of interest.

## Supporting information


**Figure S1:** Distribution histograms of activation energy (*E*_*a*_) of (i) SOM chemical oxidation (analyzed by thermal analysis as pure chemical oxidation of the whole SOM); (ii) SOM microbial mineralization (analyzed by CO_2_ efflux from soil); (iii) enzyme‐catalyzed hydrolysis of polymers and cleavage of mineral ions (analyzed by potential enzyme activity).
**Figure S2:** Activation energy (*E*_*a*_) of the chemical mineralization of SOM, analyzed by thermal oxidation (complete combustion) and by pyrolysis. Means and standard errors are presented. Numbers above each approach: the number of individual *E*_*a*_ values.
**Figure S3:** Activation energy (*E*_*a*_, mean ± SE) of the chemical mineralization of organic substances based on thermal analysis of plant and microalga organic matter. The numbers above each organic substrate reflect the number of individual *E*_*a*_ values from an additional data set for plant and microalga organic matter that do not belong to the database of *E*_*a*_ of SOM transformation. Note that the carbohydrates presented here are a wide category of simple sugars (monosaccharides), and their di‐ and oligomers are taken mainly from algae and cyanobacteria (Table S1; (Markou et al. 2012)).
**Figure S4:** Abiotic mineralization (incineration) of organic matter of plants by very intensive UV radiation in Atacama Desert.
**Table S1:** The 73 publications included in this meta‐analysis.

## Data Availability

The dataset used in this manuscript is openly available in figshare at https://doi.org/10.6084/m9.figshare.29949164.v1.
