# Supplementary material for: Activation Energy of Organic Matter Decomposition in Soil and Consequences of Global Warming
Source: Glob Chang Biol. 2025 Sep 4;31(9):e70472. doi: 10.1111/gcb.70472 (PMC12410053; doi:10.1111/gcb.70472)
Supplement: Supplementary file 1 — Figure S1: Distribution histograms of activation energy (E a ) of (i) SOM chemical oxidation (analyzed by thermal analysis as pure chemical oxidation of the whole SOM); (ii) SOM microbial mineralization (analyzed by CO2 efflux from soil); (iii) enzyme‐catalyzed hydrolysis of polymers and cleavage of mineral ions (analyzed by potential enzyme activity). Figure S2: Activation energy (E a ) of the chemical mineralization of SOM, analyzed by thermal oxidation (complete combustion) and by pyrolysis. Means and standard errors are presented. Numbers above each approach: the number of individual E a values. Figure S3: Activation energy (E a , mean ± SE) of the chemical mineralization of organic substances based on thermal analysis of plant and microalga organic matter. The numbers above each organic substrate reflect the number of individual E a values from an additional data set for plant and microalga organic matter that do not belong to the database of E a of SOM transformation. Note that the carbohydrates presented here are a wide category of simple sugars (monosaccharides), and their di‐ and oligomers are taken mainly from algae and cyanobacteria (Table S1; (Markou et al. 2012)). Figure S4: Abiotic mineralization (incineration) of organic matter of plants by very intensive UV radiation in Atacama Desert. Table S1: The 73 publications included in this meta‐analysis. [file GCB-31-e70472-s001.docx]

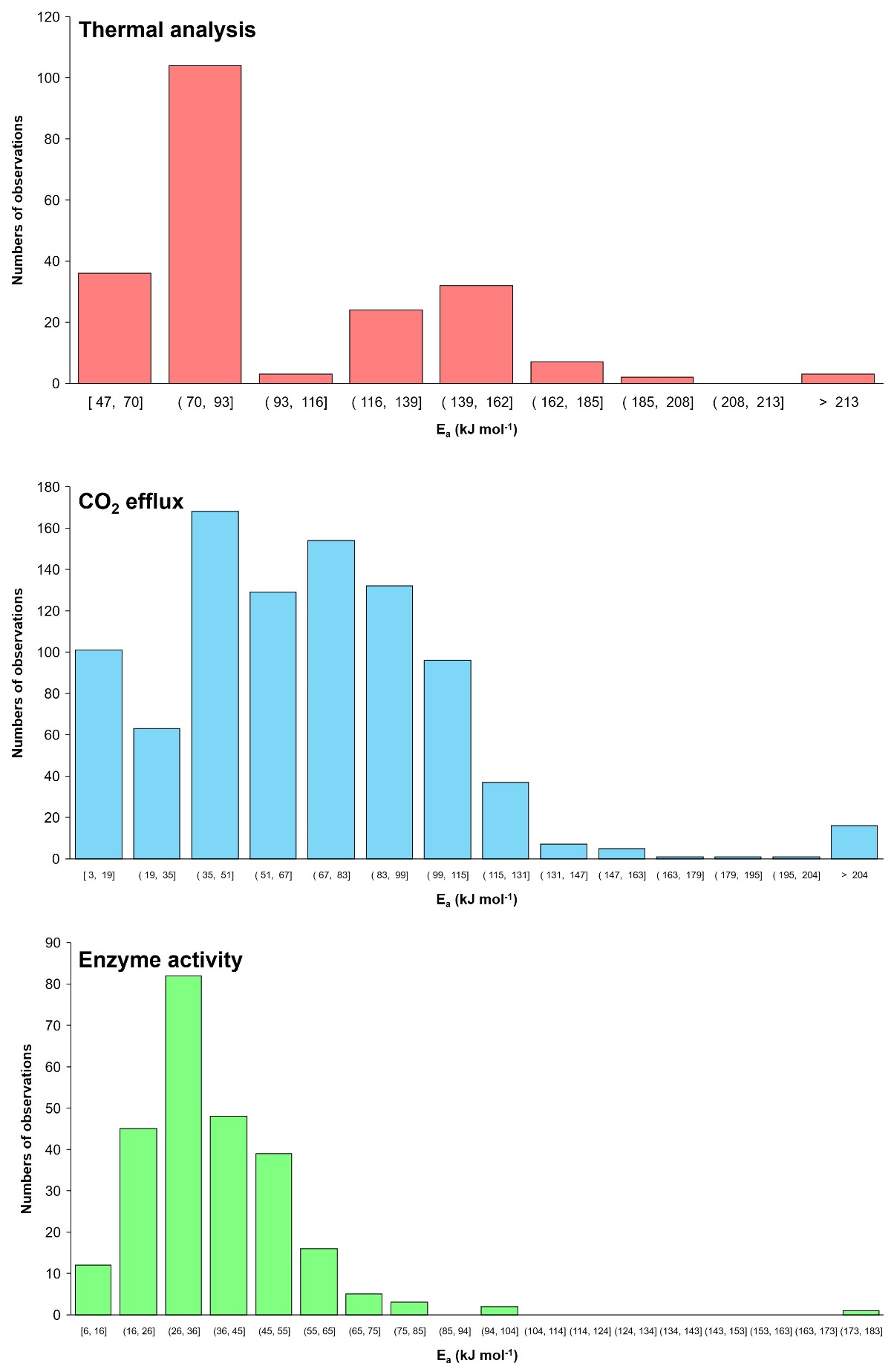


Figure S1. Distribution histograms of activation energy (E_a_) of (i) SOM chemical oxidation (analyzed by thermal analysis as pure chemical oxidation of the whole SOM); (ii) SOM microbial mineralization (analyzed by CO_2_ efflux from soil); (iii) enzyme-catalyzed hydrolysis of polymers and cleavage of mineral ions (analyzed by potential enzyme activity).


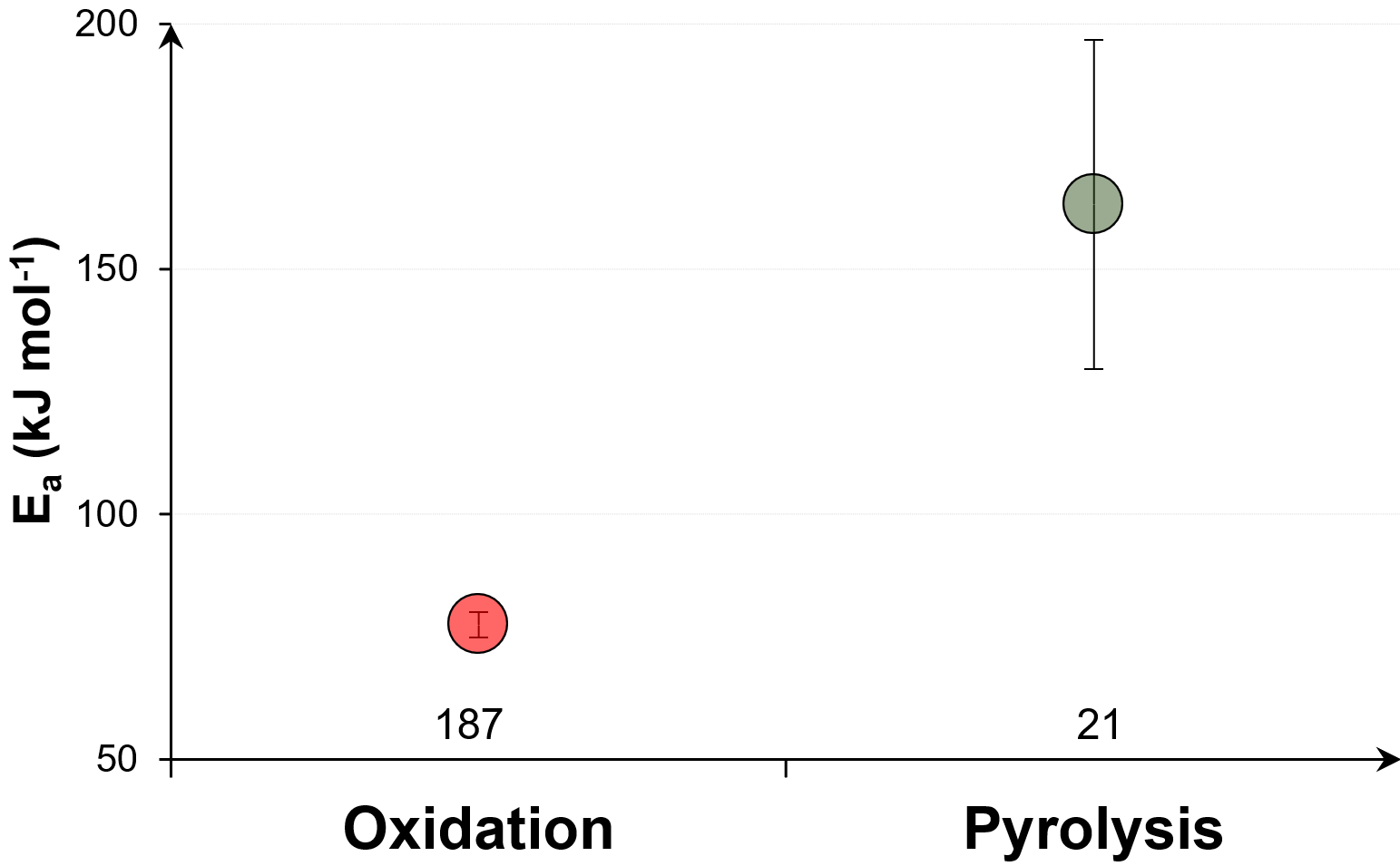


Figure S2. Activation energy (E_a_) of the chemical mineralization of SOM, analyzed by thermal oxidation (complete combustion) and by pyrolysis. Means and standard errors are presented. Numbers above each approach: the number of individual E_a_ values.


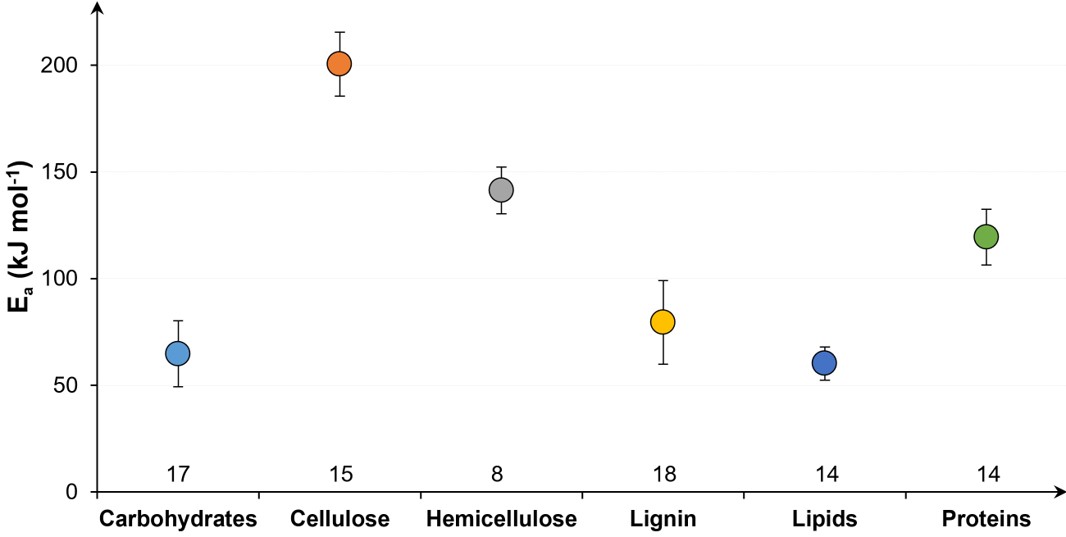


Figure S3. Activation energy (E_a_, mean ± SE) of the chemical mineralization of organic substances based on thermal analysis of plant and microalga organic matter. The numbers above each organic substrate reflect the number of individual E_a_ values from an additional data set for plant and microalga organic matter that do not belong to the database of E_a_ of SOM transformation. Note that the carbohydrates presented here are a wide category of simple sugars (monosaccharides), and their di- and oligomers are taken mainly from algae and cyanobacteria (Table S1; (Markou et al., 2012)).


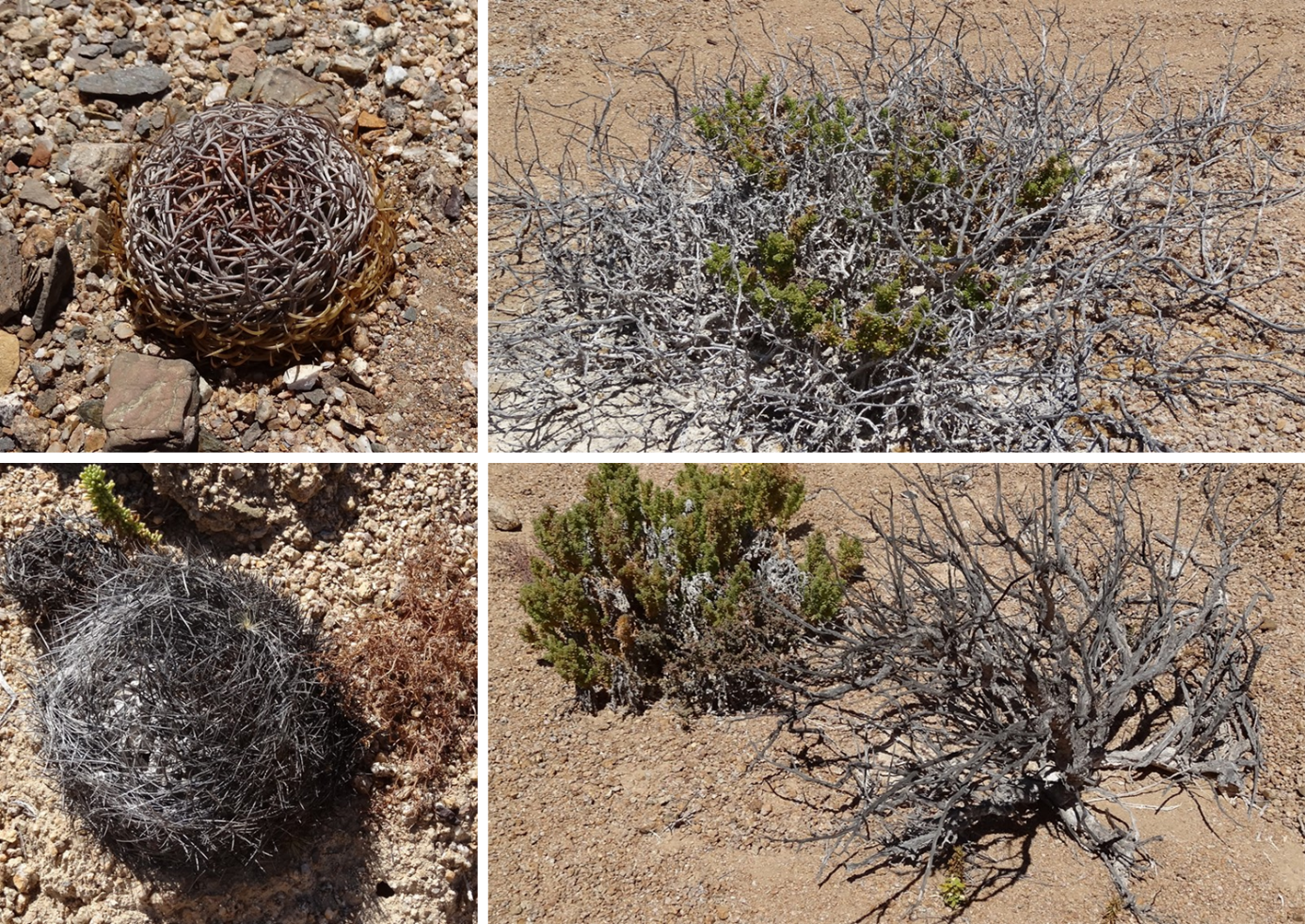


Figure S4. Abiotic mineralization (incineration) of organic matter of plants by very intensive UV radiation in Atacama Desert.

**Table S1** The 73 publications included in this meta-analysis.

1. Abdalla, M., Jones, M., Smith, P., Williams, M., 2009. Nitrous oxide fluxes and denitrification sensitivity to temperature in Irish pasture soils. Soil Use Manag. 25, 376–388. https://doi.org/10.1111/j.1475-2743.2009.00237.x

2. Acosta-Martínez, V., 2002. Inhibition of arylamidase activity in soils by toluene. Soil Biol. Biochem. 34, 229–237. https://doi.org/10.1016/S0038-0717(01)00177-8

3. Adekanmbi, A.A., Dale, L., Shaw, L., Sizmur, T., 2023. Differential temperature sensitivity of intracellular metabolic processes and extracellular soil enzyme activities. Biogeosciences 20, 2207–2219. https://doi.org/10.5194/bg-20-2207-2023

4. Alster, C.J., Baas, P., Wallenstein, M.D., Johnson, N.G., von Fischer, J.C., 2016. Temperature Sensitivity as a Microbial Trait Using Parameters from Macromolecular Rate Theory. Front. Microbiol. 7. https://doi.org/10.3389/fmicb.2016.01821

5. Alvarez, G., Shahzad, T., Andanson, L., Bahn, M., Wallenstein, M.D., Fontaine, S., 2018. Catalytic power of enzymes decreases with temperature: New insights for understanding soil C cycling and microbial ecology under warming. Glob. Change Biol. 24, 4238–4250. https://doi.org/10.1111/gcb.14281

6. An, Z., Pokharel, P., Plante, A.F., Bork, E.W., Carlyle, C.N., Williams, E.K., Chang, S.X., 2023. Soil organic matter stability in forest and cropland components of two agroforestry systems in western Canada. Geoderma 433, 116463. https://doi.org/10.1016/j.geoderma.2023.116463

7. Barja, M.I., Proupin, J., Núnez, L., 1997. Microcalorimetric study of the effect of temperature on microbial activity in soils. Thermochim. Acta 303, 155–159. https://doi.org/10.1016/S0040-6031(97)00262-1

8. Barros, N., Hansen, L.D., Piñeiro, V., Vikegard, P., 2016. Calorimetry measures the response of soil organic matter biodegradation to increasing temperature. J. Therm. Anal. Calorim. 123, 2397–2403. https://doi.org/10.1007/s10973-015-4947-8

9. Barros, N., Piñeiro, V., Hansen, L.D., 2015. Calorespirometry: A novel tool to assess the effect of temperature on soil organic matter decomposition. Thermochim. Acta 618, 15–17. https://doi.org/10.1016/j.tca.2015.09.005

10. Bárta, J., Šlajsová, P., Tahovská, K., Picek, T., Šantrůčková, H., 2014. Different temperature sensitivity and kinetics of soil enzymes indicate seasonal shifts in C, N and P nutrient stoichiometry in acid forest soil. Biogeochemistry 117, 525–537. https://doi.org/10.1007/s10533-013-9898-1

11. Bilen, S., Dick, W.A., 2011. Sulfite oxidase enzyme activity in soil. Biol. Fertil. Soils 47, 647–654. https://doi.org/10.1007/s00374-011-0564-2

12. Blagodatskaya, Е., Zheng, X., Blagodatsky, S., Wiegl, R., Dannenmann, M., Butterbach-Bahl, K., 2014. Oxygen and substrate availability interactively control the temperature sensitivity of CO2 and N2O emission from soil. Biol. Fertil. Soils 50, 775–783. https://doi.org/10.1007/s00374-014-0899-6

13. Brzostek, E.R., Finzi, A.C., 2012. Seasonal variation in the temperature sensitivity of proteolytic enzyme activity in temperate forest soils. J. Geophys. Res. Biogeosciences 117, 2011JG001688. https://doi.org/10.1029/2011JG001688

14. Chen, G., Fang, Y., Van Zwieten, L., Xuan, Y., Tavakkoli, E., Wang, X., Zhang, R., 2021. Priming, stabilization and temperature sensitivity of native SOC is controlled by microbial responses and physicochemical properties of biochar. Soil Biol. Biochem. 154, 108139. https://doi.org/10.1016/j.soilbio.2021.108139

15. Craine, J., Spurr, R., McLauchlan, K., Fierer, N., 2010. Landscape-level variation in temperature sensitivity of soil organic carbon decomposition. Soil Biol. Biochem. 42, 373–375. https://doi.org/10.1016/j.soilbio.2009.10.024

16. Craine, J.M., Fierer, N., McLauchlan, K.K., 2010. Widespread coupling between the rate and temperature sensitivity of organic matter decay. Nat. Geosci. 3, 854–857. https://doi.org/10.1038/ngeo1009

17. Craine, J.M., Gelderman, T.M., 2011. Soil moisture controls on temperature sensitivity of soil organic carbon decomposition for a mesic grassland. Soil Biol. Biochem. 43, 455–457. https://doi.org/10.1016/j.soilbio.2010.10.011

18. Dong, R., Abdelkerim-Ouba, D., Liu, D., Ma, X., Wang, S., 2023. Impacts of Partial Substitution of Chemical Fertilizer with Organic Manure on the Kinetic and Thermodynamic Characteristics of Soil β–Glucosidase. Agronomy 13, 1065. https://doi.org/10.3390/agronomy13041065

19. Dossou-Yovo, W., Parent, S.-É., Ziadi, N., Parent, L.E., 2023. CO2 Emissions in Layered Cranberry Soils under Simulated Warming. Soil Syst. 7, 3. https://doi.org/10.3390/soilsystems7010003

20. Dutta, A., Bhattacharyya, R., Jiménez-Ballesta, R., Dey, A., Saha, N.D., Kumar, S., Nath, C.P., Prakash, V., Jatav, S.S., Patra, A., 2023. Conventional and Zero Tillage with Residue Management in Rice–Wheat System in the Indo-Gangetic Plains: Impact on Thermal Sensitivity of Soil Organic Carbon Respiration and Enzyme Activity. Int. J. Environ. Res. Public. Health 20, 810. https://doi.org/10.3390/ijerph20010810

21. Elsgaard, L., Vinther, F.P., 2004. Modeling of the fine‐scale temperature response of arylsulfatase activity in soil. J. Plant Nutr. Soil Sci. 167, 196–201. https://doi.org/10.1002/jpln.200321224

22. Frankenberger, W.T., Johanson, J.B., 1983. Factors affecting invertase activity in soils. Plant Soil 74, 313–323. https://doi.org/10.1007/BF02181349

23. Frankenberger, W.T., Tabatabai, M.A., 1991. l-Asparaginase activity of soils. Biol. Fertil. Soils 11, 6–12. https://doi.org/10.1007/BF00335826

24. Funakawa, S., Nakamura, I., Akshalov, K., Kosaki, T., 2004. Soil organic matter dynamics under grain farming in Northern Kazakhstan. Soil Sci. Plant Nutr. 50, 1211–1218. https://doi.org/10.1080/00380768.2004.10408596

25. Gao, Y., Dias Da Silva, P., Alvarez, P.J.J., Zygourakis, K., 2021. Integrating Thermal Analysis and Reaction Modeling for Rational Design of Pyrolytic Processes to Remediate Soils Contaminated with Heavy Crude Oil. Environ. Sci. Technol. 55, 11987–11996. https://doi.org/10.1021/acs.est.1c03607

26. Ghosh, A., Bhattacharyya, R., Dey, A., Dwivedi, B.S., Meena, M.C., Manna, M.C., Agnihortri, R., 2019. Long-term fertilisation impact on temperature sensitivity of aggregate associated soil organic carbon in a sub-tropical inceptisol. Soil Tillage Res. 195, 104369. https://doi.org/10.1016/j.still.2019.104369

27. Ghosh, A., Das, A., Das, D., Ray, P., Bhattacharyya, R., Biswas, D.R., Biswas, S.S., 2020. Contrasting land use systems and soil organic matter quality and temperature sensitivity in North Eastern India. Soil Tillage Res. 199, 104573. https://doi.org/10.1016/j.still.2020.104573

28. Grant, K.E., Galy, V.V., Chadwick, O.A., Derry, L.A., 2019. Thermal oxidation of carbon in organic matter rich volcanic soils: insights into SOC age differentiation and mineral stabilization. Biogeochemistry 144, 291–304. https://doi.org/10.1007/s10533-019-00586-1

29. Hansen, L.D., Barros, N., Transtrum, M.K., Rodríguez-Añón, J.A., Proupín, J., Piñeiro, V., Arias-González, A., Gartzia, N., 2018. Effect of extreme temperatures on soil: A calorimetric approach. Thermochim. Acta 670, 128–135. https://doi.org/10.1016/j.tca.2018.10.010

30. He, P., Li, L.-J., Dai, S.-S., Guo, X.-L., Nie, M., Yang, X., Kuzyakov, Y., 2024. Straw addition and low soil moisture decreased temperature sensitivity and activation energy of soil organic matter. Geoderma 442, 116802. https://doi.org/10.1016/j.geoderma.2024.116802

31. Hemingway, J.D., Rothman, D.H., Grant, K.E., Rosengard, S.Z., Eglinton, T.I., Derry, L.A., Galy, V.V., 2019. Mineral protection regulates long-term global preservation of natural organic carbon. Nature 570, 228–231. https://doi.org/10.1038/s41586-019-1280-6

32. Holtan-Hartwig, L., Dörsch, P., Bakken, L.R., 2002. Low temperature control of soil denitrifying communities: kinetics of N2O production and reduction. Soil Biol. Biochem. 34, 1797–1806. https://doi.org/10.1016/S0038-0717(02)00169-4

33. Hopkins, D.W., Sparrow, A.D., Elberling, B., Gregorich, E.G., Novis, P.M., Greenfield, L.G., Tilston, E.L., 2006. Carbon, nitrogen and temperature controls on microbial activity in soils from an Antarctic dry valley. Soil Biol. Biochem. 38, 3130–3140. https://doi.org/10.1016/j.soilbio.2006.01.012

34. Joergensen, R.G., Brookes, P.C., Jenkinson, D.S., 1990. Survival of the soil microbial biomass at elevated temperatures. Soil Biol. Biochem. 22, 1129–1136. https://doi.org/10.1016/0038-0717(90)90039-3

35. Khadem, A., Raiesi, F., 2019. Response of soil alkaline phosphatase to biochar amendments: Changes in kinetic and thermodynamic characteristics. Geoderma 337, 44–54. https://doi.org/10.1016/j.geoderma.2018.09.001

36. Lee, C.G., Suzuki, S., Inubushi, K., 2018. Temperature sensitivity of anaerobic labile soil organic carbon decomposition in brackish marsh. Soil Sci. Plant Nutr. 64, 443–448. https://doi.org/10.1080/00380768.2018.1464374

37. Lefèvre, R., Barré, P., Moyano, F.E., Christensen, B.T., Bardoux, G., Eglin, T., Girardin, C., Houot, S., Kätterer, T., van Oort, F., Chenu, C., 2014. Higher temperature sensitivity for stable than for labile soil organic carbon – Evidence from incubations of long‐term bare fallow soils. Glob. Change Biol. 20, 633–640. https://doi.org/10.1111/gcb.12402

38. Leifeld, J., von Lützow, M., 2014. Chemical and microbial activation energies of soil organic matter decomposition. Biol. Fertil. Soils 50, 147–153. https://doi.org/10.1007/s00374-013-0822-6

39. Li, J., He, N., Wei, X., Gao, Y., Zuo, Y., 2015. Changes in Temperature Sensitivity and Activation Energy of Soil Organic Matter Decomposition in Different Qinghai-Tibet Plateau Grasslands. PLOS ONE 10, e0132795. https://doi.org/10.1371/journal.pone.0132795

40. Li, T., Song, F., Zhang, J., Liu, S., Xing, B., Bai, Y., 2020. Pyrolysis characteristics of soil humic substances using TG-FTIR-MS combined with kinetic models. Sci. Total Environ. 698, 134237. https://doi.org/10.1016/j.scitotenv.2019.134237

41. Liu, Y., He, N., Wen, X., Yu, G., Gao, Y., Jia, Y., 2016. Patterns and regulating mechanisms of soil nitrogen mineralization and temperature sensitivity in Chinese terrestrial ecosystems. Agric. Ecosyst. Environ. 215, 40–46. https://doi.org/10.1016/j.agee.2015.09.012

42. Ma, X., Deng, S., 2020. Quantifying the activity of β-D-fucosidase in soil. Biol. Fertil. Soils 56, 1037–1046. https://doi.org/10.1007/s00374-020-01482-9

43. Maag, M., Malinovsky, M., Nielsen, S.M., 1997. Kinetics and Temperature Dependence of Potential Denitrification in Riparian Soils. J. Environ. Qual. 26, 215–223. https://doi.org/10.2134/jeq1997.00472425002600010031x

44. Machuca, Á., Córdova, C., Stolpe, N.B., Barrera, J.A., Chávez, D., Almendras, K., Bonilla, A.M., 2018. In vitro sensitivity of forest soil enzymes to temperature increase in Western Patagonia. J. Soil Sci. Plant Nutr. 0–0. https://doi.org/10.4067/S0718-95162018005000801

45. Majeed, Z., Ajab, Z., Guan, Q., Abbasi, A.Z., Mahmood, Q., Mahnashi, M.H., Alyami, B.A.A., Alqarni, A.O., Alqahtani, Y.S., Mansor, N., 2021. Reduction in lignin peroxidase activity revealed by effects of lignin content in urea crosslinked starch under aerobic biodegradation in soil. BioResources 16, 1940–1948. https://doi.org/10.15376/biores.16.1.1940-1948

46. Matsuoka-Uno, C., Uno, T., Tajima, R., Ito, T., Saito, M., 2022. Liming and Phosphate Application Influence Soil Carbon and Nitrogen Mineralization Differently in Response to Temperature Regimes in Allophanic Andosols. Agriculture 12, 142. https://doi.org/10.3390/agriculture12020142

47. Okello, J., Bauters, M., Verbeeck, H., Bodé, S., Kasenene, J., Françoys, A., Engelhardt, T., Butterbach-Bahl, K., Kiese, R., Boeckx, P., 2022. Temperature sensitivity of soil organic carbon respiration along the Rwenzori montane forests elevational transect in Uganda (preprint). Biogeochemistry: Soils. https://doi.org/10.5194/bg-2022-37

48. Parent, L.E., MacKENZIE, A.F., 1985. RATE OF PYROPHOSPHATE HYDROLYSIS IN ORGANIC SOILS. Can. J. Soil Sci. 65, 497–506. https://doi.org/10.4141/cjss85-053

49. Parker, L.W., Miller, J., Steinberger, Y., Whitford, W.G., 1983. Soil respiration in a chihuahuan desert rangeland. Soil Biol. Biochem. 15, 303–309. https://doi.org/10.1016/0038-0717(83)90075-5

50. Paz-Ferreiro, J., Fu, S., Méndez, A., Gascó, G., 2015. Biochar modifies the thermodynamic parameters of soil enzyme activity in a tropical soil. J. Soils Sediments 15, 578–583. https://doi.org/10.1007/s11368-014-1029-7

51. Pei, J., Zhuang, S., Cui, J., Li, J., Li, B., Wu, J., Fang, C., 2017. Biochar decreased the temperature sensitivity of soil carbon decomposition in a paddy field. Agric. Ecosyst. Environ. 249, 156–164. https://doi.org/10.1016/j.agee.2017.08.029

52. Ramesh, T., Manjaiah, K.M., Tomar, J.M.S., Ngachan, S.V., 2013. Effect of multipurpose tree species on soil fertility and CO2 efflux under hilly ecosystems of Northeast India. Agrofor. Syst. 87, 1377–1388. https://doi.org/10.1007/s10457-013-9645-6

53. Razavi, B.S., Blagodatskaya, E., Kuzyakov, Y., 2015. Nonlinear temperature sensitivity of enzyme kinetics explains canceling effect—a case study on loamy haplic Luvisol. Front. Microbiol. 6. https://doi.org/10.3389/fmicb.2015.01126

54. Razavi, B.S., Liu, S., Kuzyakov, Y., 2017. Hot experience for cold-adapted microorganisms: Temperature sensitivity of soil enzymes. Soil Biol. Biochem. 105, 236–243. https://doi.org/10.1016/j.soilbio.2016.11.026

55. Reynolds, L.L., Lajtha, K., Bowden, R.D., Johnson, B.R., Bridgham, S.D., 2017. The carbon quality-temperature hypothesis does not consistently predict temperature sensitivity of soil organic matter mineralization in soils from two manipulative ecosystem experiments. Biogeochemistry 136, 249–260. https://doi.org/10.1007/s10533-017-0384-z

56. Sandeep, S., Manjaiah, K.M., Mayadevi, M.R., Singh, A.K., 2016. Monitoring temperature sensitivity of soil organic carbon decomposition under maize–wheat cropping systems in semi-arid India. Environ. Monit. Assess. 188, 451. https://doi.org/10.1007/s10661-016-5455-4

57. Sanderman, J., Grandy, A.S., 2020. Ramped thermal analysis for isolating biologically meaningful soil organic matter fractions with distinct residence times. SOIL 6, 131–144. https://doi.org/10.5194/soil-6-131-2020

58. Sokolov, D.A., Dmitrevskaya, I.I., Pautova, N.B., Lebedeva, T.N., Chernikov, V.A., Semenov, V.M., 2021. A Study of Soil Organic Matter Stability Using Derivatography and Long-Term Incubation Methods. Eurasian Soil Sci. 54, 487–498. https://doi.org/10.1134/S1064229321040141

59. Steinweg, J.M., Jagadamma, S., Frerichs, J., Mayes, M.A., 2013. Activation Energy of Extracellular Enzymes in Soils from Different Biomes. PLoS ONE 8, e59943. https://doi.org/10.1371/journal.pone.0059943

60. Stoner, S., Trumbore, S.E., González-Pérez, J.A., Schrumpf, M., Sierra, C.A., Hoyt, A.M., Chadwick, O., Doetterl, S., 2023. Relating mineral–organic matter stabilization mechanisms to carbon quality and age distributions using ramped thermal analysis. Philos. Trans. R. Soc. Math. Phys. Eng. Sci. 381, 20230139. https://doi.org/10.1098/rsta.2023.0139

61. Stoner, S.W., Schrumpf, M., Hoyt, A., Sierra, C.A., Doetterl, S., Galy, V., Trumbore, S., 2023. How well does ramped thermal oxidation quantify the age distribution of soil carbon? Assessing thermal stability of physically and chemically fractionated soil organic matter. Biogeosciences 20, 3151–3163. https://doi.org/10.5194/bg-20-3151-2023

62. Tilston, E.L., Sparrman, T., Öquist, M.G., 2010. Unfrozen water content moderates temperature dependence of sub-zero microbial respiration. Soil Biol. Biochem. 42, 1396–1407. https://doi.org/10.1016/j.soilbio.2010.04.018

63. Trasar-Cepeda, C., Gil-Sotres, F., Leirós, M.C., 2007. Thermodynamic parameters of enzymes in grassland soils from Galicia, NW Spain. Soil Biol. Biochem. 39, 311–319. https://doi.org/10.1016/j.soilbio.2006.08.002

64. Triebwasser-Freese, D.J., Tharayil, N., Preston, C.M., Gerard, P.G., 2015. Catalytic kinetics and activation energy of soil peroxidases across ecosystems of differing lignin chemistries. Biogeochemistry 124, 113–129. https://doi.org/10.1007/s10533-015-0086-3

65. Wagai, R., Kishimoto-Mo, A.W., Yonemura, S., Shirato, Y., Hiradate, S., Yagasaki, Y., 2013. Linking temperature sensitivity of soil organic matter decomposition to its molecular structure, accessibility, and microbial physiology. Glob. Change Biol. 19, 1114–1125. https://doi.org/10.1111/gcb.12112

66. Wang, G., Post, W.M., Mayes, M.A., Frerichs, J.T., Sindhu, J., 2012. Parameter estimation for models of ligninolytic and cellulolytic enzyme kinetics. Soil Biol. Biochem. 48, 28–38. https://doi.org/10.1016/j.soilbio.2012.01.011

67. Wang, Q., Wang, D., Wen, X., Yu, G., He, N., Wang, R., 2015. Differences in SOM Decomposition and Temperature Sensitivity among Soil Aggregate Size Classes in a Temperate Grasslands. PLOS ONE 10, e0117033. https://doi.org/10.1371/journal.pone.0117033

68. Wijanarko, A., 2015. Effect of organic matter and soil fertility on nitrogen mineralization and its uptake by cassava (Manihot esculenta Crantz) in a typic Hapludults. J. Exp. Biol. Agric. Sci. 3, 232–240. https://doi.org/10.18006/2015.3(3).232.240

69. Williams, E.K., Fogel, M.L., Berhe, A.A., Plante, A.F., 2018. Distinct bioenergetic signatures in particulate versus mineral-associated soil organic matter. Geoderma 330, 107–116. https://doi.org/10.1016/j.geoderma.2018.05.024

70. Williams, E.K., Plante, A.F., 2018. A Bioenergetic Framework for Assessing Soil Organic Matter Persistence. Front. Earth Sci. 6, 143. https://doi.org/10.3389/feart.2018.00143

71. Xue, J., Zhang, H., He, N., Gan, Y., Wen, X., Li, J., Zhang, X., Fu, P., 2015. Responses of SOM decomposition to changing temperature in Zoige alpine wetland, China. Wetl. Ecol. Manag. 23, 977–987. https://doi.org/10.1007/s11273-015-9434-2

72. Yanni, S.F., Diochon, A., Helgason, B.L., Ellert, B.H., Gregorich, E.G., 2018. Temperature response of plant residue and soil organic matter decomposition in soil from different depths: Decomposition with temperature and depth. Eur. J. Soil Sci. 69, 325–335. https://doi.org/10.1111/ejss.12508

73. Zhao, Z., Wu, Y., Chen, W., Sun, W., Wang, Z., Liu, G., Xue, S., 2023. Soil enzyme kinetics and thermodynamics in response to long-term vegetation succession. Sci. Total Environ. 882, 163542. https://doi.org/10.1016/j.scitotenv.2023.163542
